# An isotopic perspective on equid selection in cult at Tell eṣ-Ṣâfi/Gath, Israel

**DOI:** 10.1371/journal.pone.0326421

**Published:** 2025-07-09

**Authors:** Elizabeth R. Arnold, Haskel J. Greenfield, Gideon Hartman, Tina L. Greenfield, Shira Albaz, Elisabetta Boaretto, Johanna Regev, Aren M. Maeir

**Affiliations:** 1 Grand Valley State University, Department of Anthropology, Allendale, Michigan, United States of America; 2 Department of Anthropology and St. Paul’s College, University of Manitoba, Winnipeg, Canada; 3 University of Connecticut, Department of Anthropology, Storrs, Connecticut, United States of America; 4 University of Winnipeg, Department of Anthropology, Winnipeg, Canada; 5 Bar-Ilan University, Martin (Szusz) Department of Land of Israel Studies and Archaeology, Ramat Gan, Israel; 6 Scientific Archaeology and Dangoor Research Accelerator Laboratory, The Weizmann Institute of Science, Rehovot, Israel; 7 Bar-Ilan University, Martin (Szusz) Department of Land of Israel Studies and Archaeology, Ramat Gan, Israel; Institute for Anthropological Research, CROATIA

## Abstract

Archaeological excavations of an Early Bronze Age III (c. 2900–2600/2550 BCE) domestic neighborhood at the site of Tell eṣ-Ṣâfi/Gath, Israel, uncovered four complete skeletons of young female donkeys that were buried immediately below house floors as ritual foundation deposits. Multi-isotope analyses (carbon, oxygen and strontium) of their teeth document that each of the donkeys was born and raised in Egypt before being brought to Tell eṣ-Ṣâfi/Gath where they were slaughtered and buried beneath house floors in a non-elite domestic neighborhood. In contrast, isotopic analysis of teeth from a single isolated donkey mandible and additional sheep and goat teeth that displayed evidence of being used for food consumption and not associated with a complete burial, identify the donkey as born and raised among local livestock in the vicinity of Tell eṣ-Ṣâfi/Gath. The intentionally buried of specifically imported and highly valued young jennies reveal what appears to be a ritually charged characteristic when constructing domestic residences at the site.

## Introduction

Archaeological excavations in an Early Bronze Age III (c. 2900–2600/2550 BCE) domestic neighborhood at the site of Tell eṣ-Ṣâfi/Gath uncovered four complete young female donkey skeletons (EQ1-EQ4) buried immediately below house floors. The first excavated donkey (EQ1 – also known as a sacrificial ass) is a clear sacrificial foundation deposit [1]. Isotopic analyses indicated that it was born and raised in Egypt before being brought to the site where it was slaughtered [2]. The isotopic study has been completed on the three additional complete donkey burials excavated at the site in 2016 (EQ2 and EQ3) and 2017 (EQ4). For comparative purposes, isotopic analyses were also conducted on a loose equid mandible that was recovered in 2017 (EQ21), as well as on livestock (sheep and goat) specimens from the same deposits at the site. In addition, bioavailable strontium from the surroundings of the site were also analyzed. The results presented here confirm exchange connections between Tell eṣ-Ṣâfi/Gath and Old Kingdom Egypt as proposed by the isotopic data from EQ1. They further reveal a decision to sacrifice young and healthy female animals, those exclusively imported from Egypt.

### Tell eṣ-Ṣâfi/Gath

**Fig 1:**
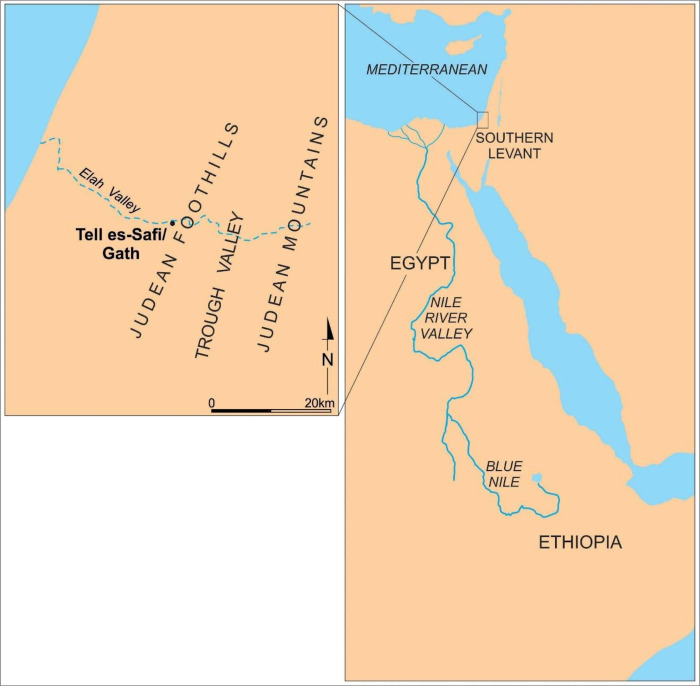
Location of Tell eṣ-Ṣâfi/Gath and regions and features (Canaan) mentioned in text.

The archaeological site of Tell eṣ-Ṣâfi/Gath (modern Tell eṣ-Ṣâfi; ancient Gath) is in central Israel about 20 km from the Mediterranean coast on the southern bank of the Elah Valley. It is located on top of a natural limestone outcrop at the westernmost edge of the Judean Foothills (*Shephelah* in Hebrew) and overlooks the modern southern Coastal Plain of Israel [3] (Fig 1). This location allowed access to fresh water and exploitation of a rich variety and abundance of natural food resources from both the rolling foothills and coastal plain [4–7]. The site is positioned along or near several major transportation routes that transect the locality to regions to the north, south, east and west. These include the north-south “Trough Valley” along the base of the Judean Mountains to the east of the site; the north-south road along the coastal plain to the west of the site; and the east-west route that extends from the coast to the Judean highlands through the Elah Valley) [8,9].

**Fig 2:**
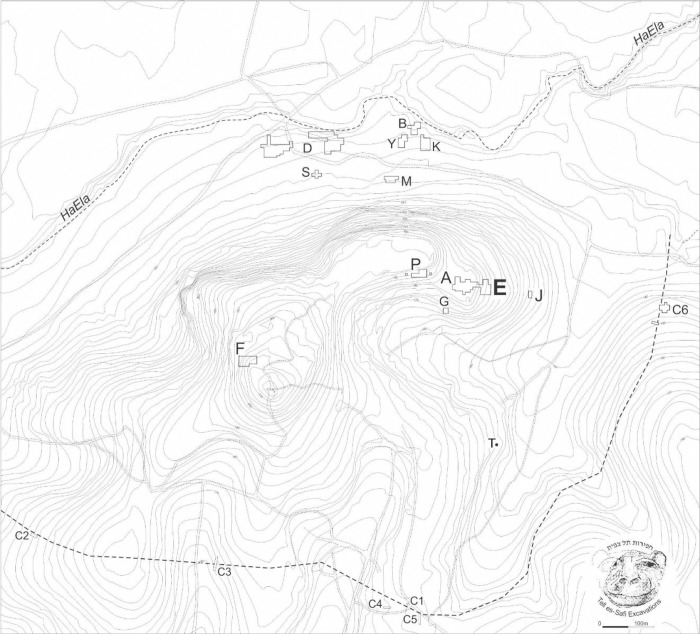
Map of Tell eṣ-Ṣâfi/Gath. Excavation areas are labeled by letters and Area E is in bold.

During the Early Bronze Age III (c. 2900–2600/2550 BCE), the site reaches urban proportions typical for this region (c. 24 ha in size) (Fig 2) and becomes one of the largest and most important of Early Bronze urban centers in the southern Levant [10–15]. It is surrounded by a thick and tall stone-based fortification system [16–24]. As a result of systematic surface collection and excavation, there is now evidence for an extensive Early Bronze (EB) occupation across the entire tell (or mound) [12,15].

Excavations conducted over the course of several seasons (2004–2017) at the eastern end of the site (Area E), uncovered part of an EB III urban residential (domestic) neighborhood [12,14]. The urban character of the site on whole during the EB III is seen through the extensive fortifications, evidence of social hierarchy and bureaucracy [25], and finds indicating economic connectivity [6,26–28] with broad regions in the Levant and beyond.

**Fig 3:**
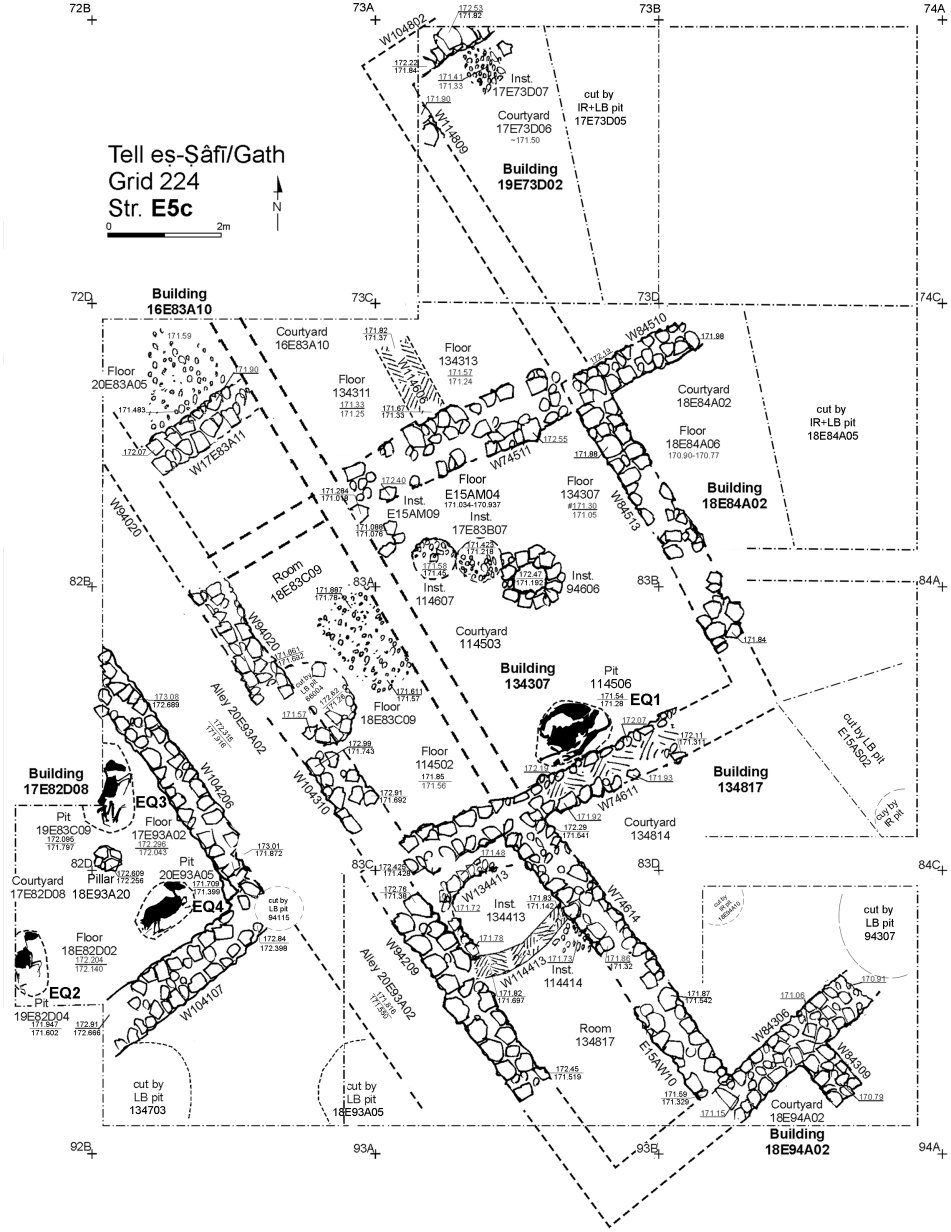
Plan of the E5c Stratum buildings in Area E at Tell eṣ-Ṣâfi/Gath with donkey burial locations.

In the excavation of Area E of the site, parts of several non-elite domestic residential buildings and an alleyway were intensively excavated and the remains studied (Fig 3). Three major strata of urban renewal occurred toward the end of the EB III period. In the earliest Stratum E5c (dated to the later EB III, c. 2700−2600 BCE), the buildings established in earlier strata (Strata E6-E7) are demolished and built anew. In the two subsequent strata (E5b and E5a), the buildings undergo further renovations where rooms are subdivided. After Stratum E5a, the site is abandoned/destroyed and there is no occupation in this area for more than a thousand years [29–31]. In the earliest E5c stratum, just before the walls were built, at least four donkeys were sacrificed and buried beneath the beaten earth floors of each building room. A thick (10−20 cm) layer of grey ashy soil accumulated above the floors during the occupation before the next strata of renewal occurred (E5b and a) [1,32–34]. Two radiocarbon contexts from Stratum E5c were measured from Building 134307, in which EQ1 was buried. The modeled 68.3% results range between 2870−2670 BCE for layer E5c, with three to four more likely ranges of years within those 200 years of probability distributions.

### The zooarchaeological sample

**Fig 4:**
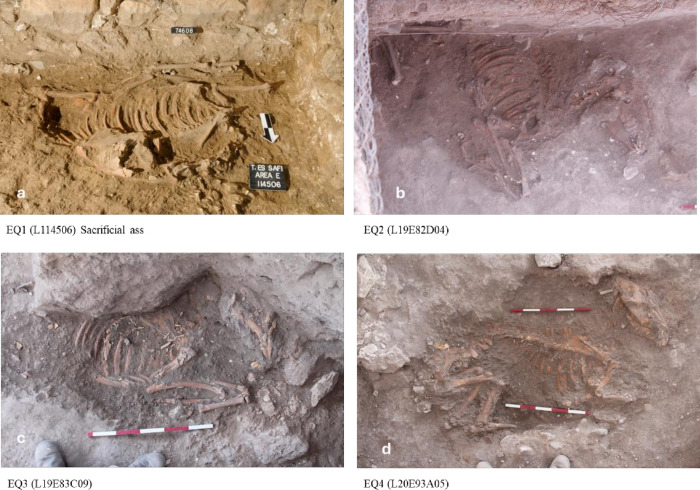
a, b, c, d. Photographs of the four donkey burials of Stratum E5c in Area E at Tell eṣ-Ṣâfi/Gath.

Four of the donkey specimens (EQ1–4) are from Stratum E5c and are completely articulated skeletons found buried beneath the earthen floor of the courtyards, at the layers of the floor infrastructure, in two large buildings on either side of the alleyway (Fig 3 and Fig 4a, b, c, d; Supplementary S1 Table). Each of the donkeys is buried in shallow pits about 20–30 cm beneath the floors in the time just before the floors were laid down as the neighborhood is being renewed. There is no evidence that they are later intrusions since they are sealed by the Stratum E5c dirt floor. All the burial pits were found without any other finds, only the equid skeletons. This contrasts with the rest of the infrastructure layers, where non-indicative pottery was found.

One skeleton lies below the floor of Building 134307 (EQ1), while three lie below the floor of Building 17E82D08 across the alley (EQ2–4). In EQ1, the front and hind legs were tied together (trussed) below the abdomen, and the upper neck (cervical) vertebra and cranium were dismembered. It is evident that the animal was sacrificed as the head is fully cut off and carefully placed on the abdomen facing in the opposite direction [1]. The head rests on its left side.

The three other complete skeletons are found across the alleyway buried beneath Courtyard 17E82D08 of Building 17E82D08. They also lie on their left sides, but without any clear evidence of dismemberment. Their legs also appear to have been trussed, as the feet are brought close together or overlap beneath their abdomens.

While there are no artefacts associated with these interments, the deposits are ritual interments [1,9], with each of the skeletons carefully laid out in the same manner. Each is placed in a separate pit with the head facing east (possibly toward the rising sun) [33]. Further, all are either old subadults or young adults and healthy females who were killed in the prime of their lives. Fragments of donkey figurine and zoomorphic vessel carrying loads were also found in Stratum E5a. That the skulls of all four donkeys face eastwards, as well as their similar age and sex, suggests a cultic/ritual choice. Such an eastern orientation of skeletal remains is similar to preferences seen in Anatolia [35] and elsewhere in the southern Levant [36].

In contrast, EQ21 (Stratum E7) is the only incomplete skeleton discussed here (L20E93A12; B20E93A139). It is represented by an isolated mandible with teeth (LPM2–4 and M1-3, but M3 is only visible in the crypt). It was found in the collapse of a Stratum E7 building amidst a concentration of stones (L20E93A11) likely representing a floor in Stratum E7. It was selected for comparison with the four donkey burials as it was an example of an equid consumed, and its remains discarded afterwards.

In addition, isotopic analysis for several sheep and goat specimens from the same horizons and bioavailable strontium specimens from surroundings of the site were also included to form a regional isoscape and baseline for comparison with the donkey remains. Each of these are discussed below to interpret the four donkeys ritually buried beneath the floors of buildings as the neighborhood is being renewed.

### Isotopic analyses in zooarchaeology

Isotope signatures are incorporated into animal tissues and are stored for variable periods of time, depending on the turnover rate of the different tissues. As tooth enamel does not turnover once formed, [37] isotopic analysis provides a picture of the animal’s diet, seasonality and/or mobility at the time of formation. Hoppe et al. [38] utilized radiographic and optical analyses to determine the timing of tooth enamel biomineralization in horses. Their results indicate that the Molar 1 (M1) begins to mineralize around two weeks of age and continues to mineralize for approximately one year after eruption (occurring between 8 months and one year). As such, the first molar records isotopic signatures of diet and mobility up to approximately two years of age. The second molar (LM2) begins to mineralize before eruption at approximately six months of age, the tooth erupts between 20–26 months and will continue to mineralize for an additional 30 months. Mineralization of molar 3 (M3) starts when M1 and M2 erupt (~6–8 months), while M3 erupts between 3.5 and 4 years. The intra-tooth sampling of teeth utilized here will reveal information on shifts in diet (carbon), water (oxygen) and mobility (strontium) from birth to approximately five years of age. As each equid is aged through tooth eruption and wear and epiphyseal fusion at approximately five years of age^1^ this sampling strategy documents their entire lifespan.

The use of stable carbon isotopes to reconstruct diet is well-established in archaeology. Terrestrial plants in the southern Levant can be divided into two groups (C_3_, C_4_) based on their differential means of fixing atmospheric CO_2_. These differential mechanisms result in distinct δ^13^C values. These differences in the δ^13^C values of the plants consumed are reflected in the tissues of the animals that consume them [39–41]. While C_3_ vegetation is dominant in Mediterranean climate zones [42], C_4_ grasses are also part of the flora of Mediterranean regions [43] and their abundance increases in steppe and desert environments [44].

Oxygen isotope composition in the tooth enamel in mammals is linked to the isotopic composition of environmental water they consume [45,46]. As the isotopic composition of the water is correlated with temperature and evapotranspiration, it will vary seasonally. In mid- to high- latitudes the lowest δ^18^O values are seen in the coldest months and the highest δ^18^O values during the summer months [47].

Strontium isotopes are useful as biogeochemical tracers in the study of mobility as they are characteristic of the local geology [48]. Strontium ^87^Sr/^86^Sr ratios vary locally with changes in local bedrock geology, bedrock weathering, and the contribution of atmospheric dust [49]. Soil and plants are in isotopic equilibrium with local soils and share similar isotopic ratios for bioavailable strontium. Animals consuming these plants incorporate strontium in the mineral structure of bones and teeth as calcium substitutes during tissue formation. Measurement of the strontium ratio will provide a measure of the relative importance of foods from areas of variable lithologies [50]. As a result, migration studies should utilize biologically available strontium rather than only substrate geology. Plants, soil, rodents and/or invertebrates may be collected for this purpose.

## Results

**Fig 5:**
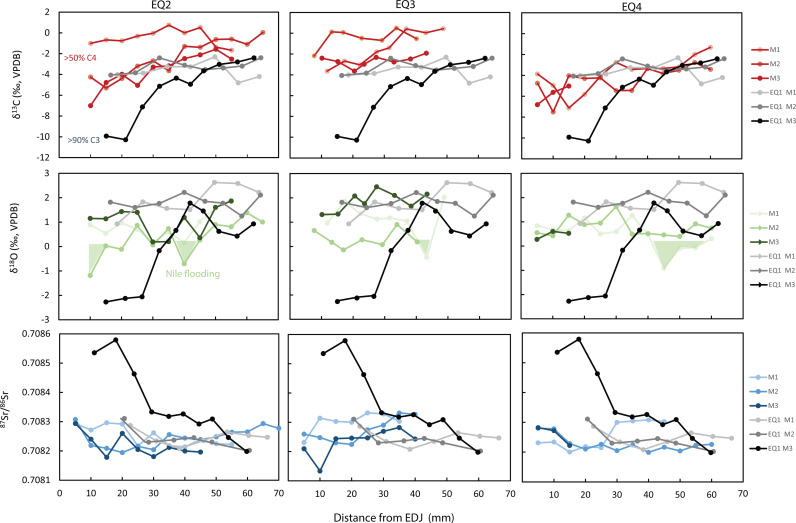
Sequential isotopic carbon, oxygen and strontium sampling of EQ2, EQ3 and EQ4 mandibular teeth from Area E of Tell eṣ-Ṣâfi/Gath.

**Fig 6:**
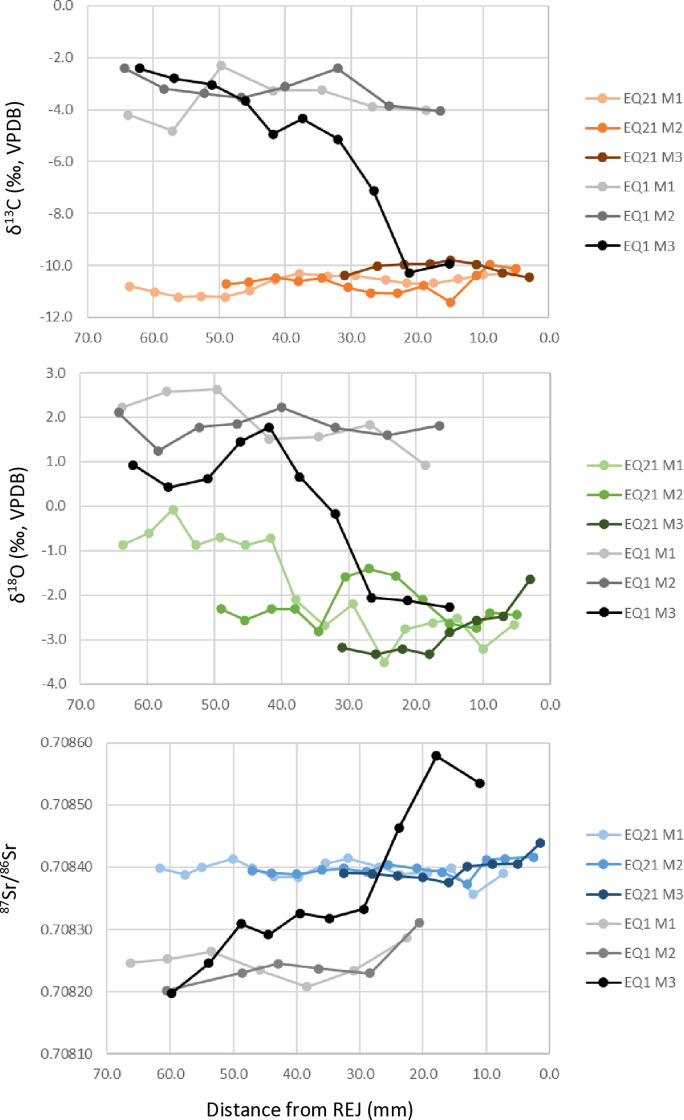
Sequential isotopic carbon, oxygen and strontium sampling of EQ21 mandibular teeth from Area E of Tell eṣ-Ṣâfi/Gath.

Isotope results are presented in Fig 5 and 6 and in Supplementary S2 and S3 Tables. Descriptive statistics are presented in Table 1.

**Table 1:** Descriptive statistics for the isotopic analysis of the Tell eṣ-Ṣâfi/Gath domestic donkeys.

			87Sr/86Sr					
Locus	Indivdual	Tooth	n	Mean	2 s.d	min	max	∆min-max
114506	EQ1*	M1	7	0.708247	0.00005	0.708208	0.708287	0.00008
114506	EQ1*	M2	6	0.708242	0.00007	0.708202	0.708245	0.00004
114506	EQ1*	M3	10	0.70836	0.00025	0.708198	0.708579	0.00038
19E82D04	EQ2	M1	11	0.70825	0.00007	0.708214	0.708296	0.00008
19E82D04	EQ2	M2	14	0.708245	0.00007	0.708194	0.708308	0.00011
19E82D04	EQ2	M3	9	0.708218	0.00008	0.708178	0.708294	0.00012
19E83C09	EQ3	M1	7	0.708302	0.00007	0.708231	0.708332	0.00010
19E83C09	EQ3	M2	8	0.708273	0.00008	0.708225	0.708332	0.00011
19E83C09	EQ3	M3	8	0.708234	0.00009	0.708134	0.708282	0.00015
20E93A05	EQ4	M1	9	0.708257	0.00009	0.7082	0.708308	0.00011
20E93A05	EQ4	M2	12	0.708227	0.00005	0.708199	0.708278	0.00008
20E93A05	EQ4	M3	3	0.708259	0.00006	0.708222	0.708282	0.00006
20E93A12	EQ5	M1	15	0.708394	0.00003	0.708356	0.708415	0.00006
20E93A12	EQ5	M2	16	0.708392	0.00003	0.70837	0.708416	0.00005
20E93A12	EQ5	M3	9	0.708397	0.00004	0.708386	0.708439	0.00005
			δ13C					
Locus	Indivdual	Tooth	n	Mean	s.d	min	max	∆min-max
114506	EQ1*	M1	7	−3.7	0.8	−4.8	−2.3	2.5
114506	EQ1*	M2	8	−3.2	0.6	−4.1	−2.4	1.7
114506	EQ1*	M3	10	−5.4	2.8	−10.3	−2.4	7.9
19E82D04	EQ2	M1	10	−0.5	0.8	−1.7	0.7	2.4
19E82D04	EQ2	M2	12	−2.4	1.7	−5.3	0	5.3
19E82D04	EQ2	M3	10	−3.6	1.6	−7	−1.6	5.4
19E83C09	EQ3	M1	8	−1.5	1.6	−3.6	0.4	4
19E83C09	EQ3	M2	7	−0.5	0.9	−2.2	0.5	2.7
19E83C09	EQ3	M3	8	−2.7	0.5	−3.6	−1.9	1.7
20E93A05	EQ4	M1	12	−3.8	1.3	−7.1	−1.3	5.8
20E93A05	EQ4	M2	12	−4.4	0.9	−7.5	−2.8	4.7
20E93A05	EQ4	M3	3	−5.8	1.6	−6.8	−5	1.8
20E93A12	EQ5	M1	16	−10.7	0.3	−11.2	−10.2	1
20E93A12	EQ5	M2	13	−10.6	0.3	−11.1	−9.9	1.2
20E93A12	EQ5	M3	8	−10.1	0.2	−10.5	−9.8	0.7
			δ18O					
Locus	Indivdual	Tooth	n	Mean	s.d	min	max	∆min-max
114506	EQ1*	M1	7	1.9	0.6	0.9	2.6	1.7
114506	EQ1*	M2	8	1.4	1.2	−1.6	2.2	3.8
114506	EQ1*	M3	10	−0.1	1.5	−2.3	1.8	4.1
19E82D04	EQ2	M1	10	0.9	0.6	0.2	1.9	1.7
19E82D04	EQ2	M2	12	0.3	0.8	−1.2	1.4	2.6
19E82D04	EQ2	M3	10	1.1	0.6	0.2	1.9	1.7
19E83C09	EQ3	M1	8	1.1	0.7	−0.5	2	2.5
19E83C09	EQ3	M2	7	0.3	0.4	−0.1	0.9	1
19E83C09	EQ3	M3	8	1.9	0.4	1.3	2.5	1.2
20E93A05	EQ4	M1	12	0.4	0.4	−0.9	1.3	2.2
20E93A05	EQ4	M2	12	0.8	0.2	0.4	1.6	1.2
20E93A05	EQ4	M3	3	0.5	0.6	0.3	0.6	0.3
20E93A12	EQ5	M1	16	−1.8	1.1	−3.5	−0.1	3.4
20E93A12	EQ5	M2	13	−2.2	0.4	−2.8	−1.4	1.4
20E93A12	EQ5	M3	8	−2.8	0.6	−3.3	−1.6	1.7

* previously published data [2].

### Carbon isotopes

EQ2, EQ3, and EQ4 (n = 9 molars; 82 samples) have mean carbon isotope values of −2.7‰ and ranges from −7.5‰ to 0.7‰ (S2 Table, Fig 5) while EQ21 (n = 3 molars; 37 samples) has a ${\;\delta }^{13}C\;\overset{―}{\underset{―}{x}}$ = −10.5‰ and a range between −11.2‰ to −9.8‰ (Fig 6). These values suggest that EQ2, EQ3 and EQ4 were eating vegetation from a mixed C_3_/C_4_ environment with a high proportion of C_4_ vegetation as was seen with EQ1 [2]. EQ21’s carbon values show a diet of >90% C_3_ from all molar samples, which indicates the dominance of C_3_ vegetation in the diet, matching the diet of previously sampled caprines (abbreviated OC for *Ovis/Capra* including both sheep and goat) [51].

### Strontium isotopes

EQ2, EQ3 and EQ4 (n = 9 teeth, 81 samples) have average strontium ratios of 0.70824, and ranges of 0.70813 to 0.70833 (S3 Table, Fig 5). There are obvious similarities to the strontium ratios of the first and second molars of EQ1. EQ21 (n = 3 teeth; 40 samples) has an average of 0.70839, and ranges between 0.70836 to 0.70844 (Fig 6).

### Oxygen isotopes

The mean δ^18^O values for EQ2, EQ3, and EQ4 (n = 9 teeth; 82 samples) ${\;\delta }^{18}O\;\overset{―}{x}$ = 0.96‰ and values range between −1.6 -to 2.6‰ (S2 Table, Fig 5). Modern oxygen isotope values for the Nile River before the construction of Aswan Dam provide an average δ^18^O (VSMOW) of 0.74‰ and measured in shallow groundwater reservoirs [52]. These values align with the mean oxygen isotope values for Egyptian archaeological human tooth and bone carbonate values analyzed at different locations along the Nile valley (1.05 ± 1.02‰) [53]. The seasonal fluctuation in the δ^18^O values of sacrificial donkeys EQ2, EQ3, and EQ4, record short negative spikes (~−1‰ relative to adjacent samples and marked by green shaded areas in Fig 5). These correspond to summer season incorporation of Blue Nile water from the Ethiopian highland with measured $\begin{array}{cc} \;\overset{―}{x}=\;-\mathrm{.70}\pm 1.05 & \#x2030;\;(VSMOW) \end{array}$ [55]. Calculating the mean δ^18^O values of all the molar teeth of EQ2, EQ3 and EQ4 excluding the negative pulses highlights the significance of these shifts. The average of EQ2 molars minus the two negative samples at 6.4 mm and 34 mm (n = 30) is 0.8 ± 0.56‰ (1 std. dev.) vs. 0.95‰, the mean of the two negative pulses, a difference more than two standard deviations lower than the average. This pattern is repeated in EQ3 (n = 22; 1.2 + 0.8‰ vs. −0.5‰) and EQ4 (n = 22; 0.7 + 0.4‰ vs. −0.4‰). The oxygen isotope values for EQ21 are more negative ${\;\delta }^{18}O\;\overset{―}{x}$ = −2.3‰ than those of the donkeys (2 tail t-test assuming equal variance p > 0.00001) and range between −0.1 to −3.5‰ (Fig 6). EQ21 values agree with oxygen isotope values measured in caprines from Tell eṣ-Ṣâfi/Gath [51] and correspond to mean modern δ^18^O values of rainfall of the southern Levant in proximity to the site between −3.5– (−5)‰ (VSMOW) [54].

### Statistical analyses

**Fig 7:**
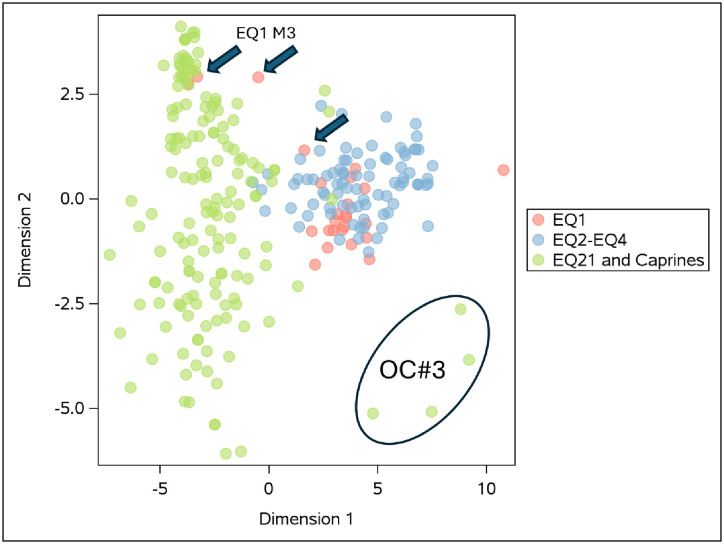
MDS Procedure plot of all domestic animals. The unique pattern of OC#3 is highlighted. Arrows indicate the final three samples of the third molar of EQ1.

The statistical procedure PROC DISTANCE in SAS was used to assess all of the isotope data (carbon, oxygen and strontium) currently available from Tell eṣ-Ṣâfi/Gath (EQ1 [2]; all caprines [51]) and the three additional equids (EQ2, EQ3, EQ4 and EQ21) discussed here. The DISTANCE procedure computes measures of dissimilarity or similarity between the observations. The output data set was then used as input to the MDS procedures. The MDS PROC performed multidimensional scaling to create Dimensions 1–4 (see Supplementary S4 Table). Dimensions 3 and 4 are all near zero and are redundant. As such, only Dimensions 1 and 2 are graphed (Fig 7).

## Discussion: Egyptian origins

Previous isotopic analyses of EQ1 utilized carbon, oxygen and strontium isotopic proxies as evidence that this individual was born and lived in the Nile Valley prior to travel and eventual sacrifice at the site of Tell eṣ-Ṣâfi/Gath [2]. EQ1 completed its early tooth development in the Nile Valley and later migrated to Tell eṣ-Ṣâfi/Gath where it completed the formation of its third molar. The three samples closest to the root/enamel junction (REJ) are the last to mineralize and reflect the shift in diet, water source and local geology (highlighted with arrows in Fig 7). This corroborates textual and other archaeological information that already pointed toward the existence of long-distance trade of donkey caravans between Egypt and Canaan during this early urban period.

The analyses of four additional equids (from 3 burials and a single loose mandible) presented here provides additional direct evidence for the import of equids from Egypt to the site of Tell eṣ-Ṣâfi/Gath. While all four burials point to an Egyptian origin, the single loose mandible isotopic signature suggests a local origin from around the site. This is mirrored by the results of the other domestic livestock and bioavailable strontium analyses. While a single caprine was also identified as having an Egyptian origin, all the other caprines sampled (n = 16) reflect discrete values that indicate their life history was focused locally at Tell eṣ-Ṣâfi/Gath [2,51].

**Fig 8:**
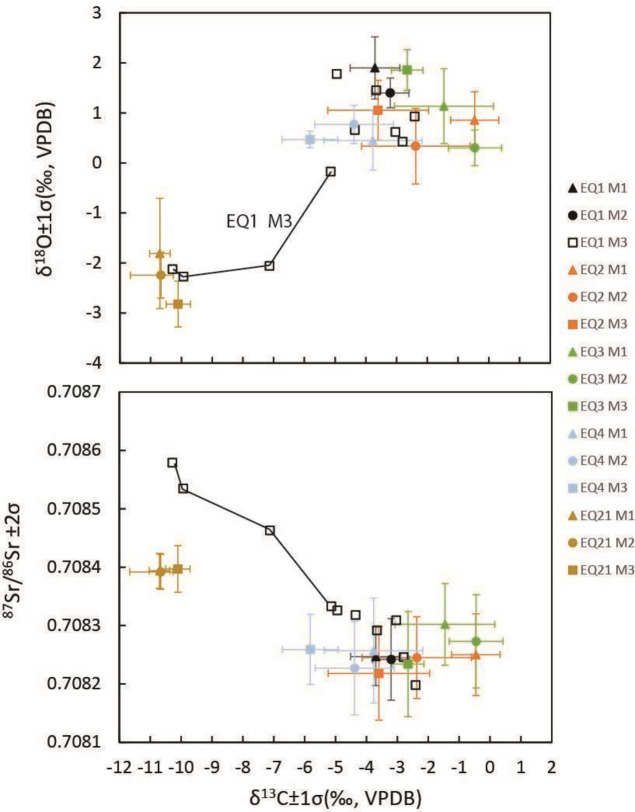
Local vs. Nile Valley bivariate plotting of mean intra and inter-tooth isotope values.

The primary indicator for Nile Valley origins of EQ1–4 is the dominance of C_4_ vegetation (>50%) in their diet, as indicated by the δ^13^C carbonate values measured in the early tooth formation of the EQ1 (M_1_ + M_2_) and in all three molar teeth of EQ2, EQ3 and EQ4 (Fig 5). As has been previously asserted [1] the Nile Valley is the nearest geographic location where C_4_ vegetation can serve as primary fodder for herbivores [55,56]. Seasonal shifts in carbon isotope values can be associated with agricultural cycles in the Nile Valley. C_4_ plants are not the sole forage or fodder source in Egypt but serve as a good food source for livestock. It is evident that on a seasonal basis donkeys might eat more C_3_ plants (by-products of agricultural production). The expected pattern of change in carbon isotopes for an animal moving from the Nile River Valley to the interior of the southern Levant would be a shift from a C_4_ plant dominant diet to a predominant C_3_ diet (Fig 8).

This pattern is reflected in all equids showing a greater C_3_ contribution to the diet in their third molars. In contrast, the δ^13^C values of the sampled caprines predominantly reflect the C_3_ vegetation that dominates the Mediterranean climate with only a minor contribution of C_4_ vegetation [51]. EQ21’s carbon values show a diet of >90% C_3_ from all molar samples which indicates the dominance of C_3_ vegetation in the diet (Fig 6).

EQ1 δ^18^O values match archaeological human tooth and bone carbonate values that were analyzed at different locations along the Nile valley (+1.05 ± 1.02‰)^54^ and the same is true for EQ2, EQ3 and EQ4. In addition, the oxygen isotopes of EQ2, EQ3 and EQ4 provide an important secondary marker for Egyptian origin with the observation of the Blue Nile negative oxygen isotope pulses along the teeth. Sequential sampling along first and second molars display a negative dip (~1‰) of oxygen isotope values at around 33−43 mm from the root-enamel-junction (REJ) that is congruent with seasonal flow of tributary water into the Nile River. These low points are associated with late summer flooding caused by the Blue Nile input. The flow of monsoon water from the highlands of Ethiopia into the Blue Nile occurs during late summer months and provides a pulse of ^18^O depleted water that dominate premodern Nile volume (85% of Nile flow) and composition [57,58]. The summer main rainy season mean oxygen isotope composition of the Blue Nile from the highlands of Ethiopia is −2.3‰ [57]. The intensity of Blue Nile water flow varies between years in agreement with ENSO (El Nino Southern Oscillation) [59]. It is therefore unsurprising to see variability in the expression of Blue Nile signal within and between individuals. Further, a second negative dip in the oxygen isotope values is suggested by trends in the final samples of the teeth indicating a complete seasonal cycle.

In contrast, the oxygen isotope values for EQ21 are more negative ${\;\delta }^{18}O\;\overset{―}{\underset{―}{x}}$ = −2.3‰ and match the δ^18^O values in caprine (OC) teeth studied at Tell eṣ-Ṣâfi/Gath [51]. Values vary between 0 – (−2) ‰ (n = 13, ${\;\delta }^{18}O\;\overset{―}{\underset{―}{x}}$ and are comparable to prehistoric gazelles originating in low topographies of the eastern Galilee and much higher than the negative values measured in high topography gazelles [60]. This pattern suggests that the EB caprine herds, and EQ21 were herded eastwards from the site to the lower reaches of the Judean Mountains. Even though the Judean Mountains elevation reaches ~1000 m above modern sea level, there is no indication that they were herded into the higher reaches of the Judean Mountains since their isotopic values are comparable to the gazelle’s low topographies values.

Arnold et al. [2] detail plant collection strategy and results that allowed creation of a local isoscape surrounding the site of Tell eṣ-Ṣâfi/Gath. There is an ^87^Sr/^86^ Sr range of 0.7084–0.7085 for the *rendzina* soils that cover the area east of the site. This hilly region is very suitable for the herding of both grazers (sheep) and browsers (goats), while the flatter dark brown alluvium vertisol soils located west of the site that extend to the seashore are more suitable for rainfed cereal agriculture [61]. Plant sampling also took place on Holocene alluvium soils (vertisols) to the west of the site. However, this sampling cannot provide a credible estimate for all alluvial soils as alluvium strontium ratios vary depending on parent bedrock weathering and atmospheric deposition. *Terra rossa* (FAO: Cambisols and Phaeozems) soils develop on hard marine sedimentary bedrock (limestone and dolomite) primarily through atmospheric deposition [49,61]. This is evident from examination of plants growing on these soils since they incorporate bioavailable strontium with ^87^Sr/^86^Sr ratios of 0.7086 ± 0.0003. Moffat et al. [62] report two soil data points in the vicinity of the site that agree with ^87^Sr/^86^Sr ratios ranging from 0.70828–0.70870.

In contrast, isoscapes are not as well developed in Egypt. A literature review of isotopic studies for the Nile Valley presents a median based on both animal and human data of 0.7076 ± 0.0003. However, the analyzed faunal samples show sizeable ranges and much wider ranges than the majority of the human samples. While the data from Tell el-Dab‘a (Hutwaret/Avaris) in the northeastern Nile Delta fits this range [63], it is problematically drawn from six archaeological bone samples. Strontium analysis from bone is problematic due to the greater impact of diagenesis on bone [64] as there is no accepted pretreatment that can fully deal with the issue of diagenesis in bone [65]. In addition, one of the Egyptian “baseline” animals is an equid, which is potentially a very mobile animal in the landscape as is argued here. Human burials from the Nile Valley yield tighter ratio ranges. For example, Memphis in Lower Egypt, $\underset{―}{x}$=0.7078 ± 0.003; range 0.7074–0.7087 [66]. Although not exclusive to the Nile Valley, the strontium ratios do not contradict the other two independent lines of isotopic evidence – carbon and oxygen, that argue for Egyptian origins of EQ2, EQ3 and EQ4.

The statistical analyses display two clusters. The first and second molars of EQ1 (while that individual lived in Egypt), EQ2, EQ3 and EQ4 all cluster on the right side of the graph (Fig 7). As does OC (Caprine) #3 – previously identified as having an Egyptian origin [2,51]. EQ21 and most caprines (apart from OC#3) all cluster on the left side of the graph. Also, on the left side of the graph, are the two samples closest to the REJ of EQ1’s M3 as these are the final samples to mineralize and thereby incorporate the isotopic signatures surrounding the site. From the pooled data of carbon, oxygen and strontium isotope values, we define a foreign cluster (EQ2, EQ3, EQ4, EQ1 first and second molars and OC#3) versus a local cluster at the site that includes EQ21, all other OC, the two final samples of EQ1’s third molar, one final sample of EQ2 and the two final samples of EQ4.

## Discussion: Selection for sacrifice

All equids imported from Egypt are buried, fully articulated in household contexts and under habitation floors. Only equids receive this treatment and only equids from Egypt. One goat indicates Egyptian origins but was not intentionally buried [51]. Rather it was butchered, eaten and its remains discarded in a midden. One donkey (EQ21) does not have isotopic values that indicate its origin in Egypt, and it was also not intentionally buried. It was butchered and its remains were discarded.

From our analysis, the donkeys imported from Egypt had greater social value than those raised and utilized locally. Cross-culturally, the selection of sacrificial animals is known to be based on criteria such as age, sex, species [67] and even coat color when preservation allows [68,69], but never seemingly selected based on geographic origin. From the perspective of the isotope studies, it is the Egyptian import status that is one of the key criteria for an animal to be selected for sacrifice. Being young and female are the other two variables. Thus, young female donkeys from Egypt are the ideal sacrificial animal at Tell eṣ-Ṣâfi/Gath during the EB III. Perhaps, as noted elsewhere, they represented a distinct totem animal linked with the identity of inhabitants as merchants/traders [9]. These stand in contrast to a local equid (EQ21 – unable to determine sex) that was butchered and likely eaten. EQ21 derives from an earlier deposit at the site (Stratum E7) which slightly pre-dates the four completely articulated equid burials (probably less than 50 years).

It is suggested that even within this preferred group, EQ1 is noteworthy. EQ1 was buried with the front and hind legs tied together (trussed) below the abdomen, and the upper neck (cervical) vertebra and cranium dismembered and placed on the abdomen facing east. It is evident the animal was sacrificed, the head entirely cut off and carefully placed on the abdomen facing in the opposite direction [1]. In contrast, the other equids were buried without trussed limbs and without decapitation. In addition, it appears that EQ1 alone was prepared for sacrifice as the isotopic data indicates her residence at the site for several months prior to sacrifice as is evident from the three final data points (on M_3_ closest to REJ) as has been previously argued [2]. These final strontium ratios are also more radiogenic than the other equids and local livestock. These ratios reflect agricultural areas on terra rosa soils like those found to the west of Tell eṣ-Ṣâfi/Gath [51]. It can be deduced that even though EQ1 was grazed locally toward the end of her life, she was treated slightly differently from the other local equids. This imported donkey was kept penned and foddered with hay that was harvested in the valley, a product of dry farmed cereals. This donkey was never herded with other livestock east of the site.

The archaeological, iconographic and textual evidence from the broader region surrounding the site of Tell eṣ-Ṣâfi/Gath highlights the centrality of equids in the culture and societies of the mid-3rd millennium BCE. Equids are known as work animals within the agricultural systems, used for plowing, threshing and as draft animals. In addition, equids played a significant role in trade, not only moving goods on their backs but also as valuable trade items themselves [33,70–72].

It is clear that some equids were elevated to the status of prestige animals as evidenced by their intentional sacrifice and burial at sites across the Levant, Syria and Mesopotamia. Notably, these burials occur in a variety of different contexts, both domestic [33] and elite [70,73,74], singularly and in pairs [75], both males and females and varying in age from nine months [75] to nine years [76]. This suggests regional preferences for specific types of equids for sacrifice, such as those from Egypt, as is proposed here. Further, the textual data also discusses selective breeding of equids and desired qualities of animals for particular purposes [73,75].

Our analysis has demonstrated that the non-local donkeys (young females in particular) were preferentially selected for sacrifice and burial beneath the floors of buildings as the neighborhood is being renewed in the Early Bronze III urban center at Tell eṣ-Ṣâfi/Gath. The isotopic analysis suggests that imported Egyptian asses were preferentially selected as sacrificial animals, while locally raised equids were butchered and likely eaten.

The data further highlights the charged ritual significance and value of select donkeys in this period of early complex state-level urban societies in the region. It was not any donkey that was deemed worthy of sacrifice at Tell eṣ-Ṣâfi/Gath, but only Egyptian young females. This suggests the function of these sacrifices is not simply a totem animal or an occupation marker but was socially and ritually charged by having come from Egypt. The importance and charged significance of the display of economic, and likely social and political connections with Egypt, was an integral part of the sacrificial ritual. Thus, an Egyptian donkey might have been seen as an exotic and special animal, worthy of specific ritual use.

## Materials and methods

All necessary permits were obtained for the study described, which complied with all relevant regulations. Excavation permits were issued to Aren Maeir by the Israel Antiquities Authority (Permit #, G-56/2008, G-51/2010, G-66/2016, G-62/2017). Permits for export of material from Israel were issued to Aren Maeir by the Israel Antiquities Authority (Permit # 13840 – July 25, 2012). Additional information regarding the ethical, cultural, and scientific considerations specific to inclusivity in global research is included in the Supporting Information (Supplementary S1 Checklist)

**Fig 9:**
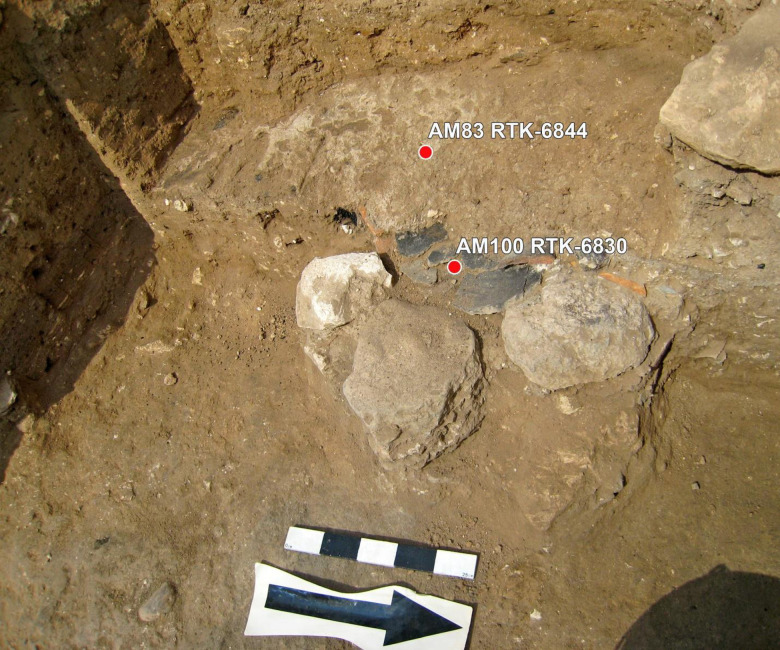
Contexts of the radiocarbon samples from stratum E5c, Ins. E15AM09.

**Fig 10:**
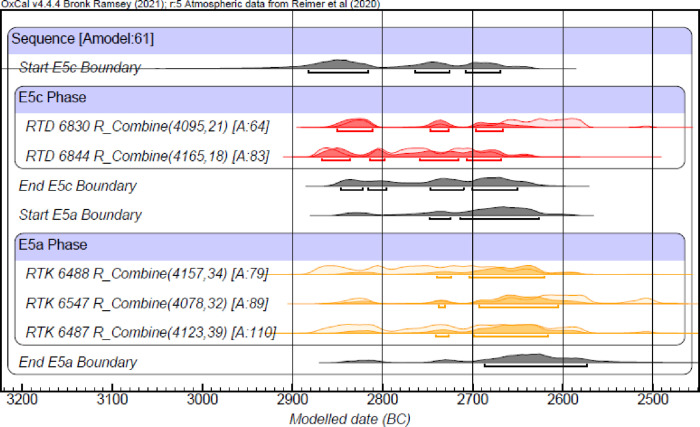
Modeled probability distributions of radiocarbon dates from layers E5c and E5a. The 68.3% probability distributions are plotted in darker color and marked by a line underneath the plots.

Two radiocarbon contexts (RTD 6830 and RTD 6844) from Stratum E5c were measured from Building 134307, in which donkey (EQ1) was buried. The samples were taken from an installation (E15AM09) (Fig 9). Sample RTD 6830 (Basket # TS12E15AM100) was taken within broken sherds of a holemouth cooking pot found inside a cooking installation built of stones. Seeds were measured in three separate targets to reach a higher precision for the context. For sample RTD 6844 (Basket # TS12E15AM083), seeds were taken from a thin white layer of phytoliths covering the cooking installation. Four separate targets were measured for a high precision date. For the sampling, pretreatment and measurement protocols [77–81] of the Dangoor Research Accelerator Spectroemter at the Weizmann Institute. The radiocarbon dates were calibrated using Oxcal v4.4.4 Bronk Ramsey (2021) software based on the calibration curve Reimer et al 2020 [81]. The measurements of each context were combined using the Rcombine function in the Oxcal v4.4.4 program, and both contexts passed the χ^2^ test. We built a small model using Oxcal v4.4.4 software [80,81] together with the samples from Stratum E5a [14], including a sequential boundary between the layers, since no samples from Stratum E5b were measured from this room. The agreement of the model is 61%. The modeled 68.3% results range between 2870−2670 BC for layer E5c, with three to four more likely ranges of years within those 200 years of probability distributions (Fig 10 and Table 2).

**Table 2:** Radiocarbon dates and their calibrated ranges from layer E5c. The Rcomb refers to the average of the multiple radiocarbon determinations for the same sample material. Modelled Rcomb refers to the calibrated range of the sample after Bayesian analysis modelling. The modelled distribution is influenced by the presence of the samples in layer E5a as shown in Fig 10 in yellow.

Laboratory #	Libby Age year BP	± 1σ	Calibrated range BC 68.3% probability	Calibrated range BC 95.4% probability	Context Description
RTD 6830.1	4090	35	2836 (11.9%) 2818 2666 (13.8%) 2646 2636 (42.6%) 2579	2851(19.3%) 2808 2747 (5.0%) 2726 2698 (70.3%) 2572 2512 (0.9%) 2505	L.E15AM08 B.TS12E15AM100 Seeds in phytoliths layer
RTD 6830.2	4100	35			
RTD 6830.3	4095	35			
Rcomb 6830 X2-Test: df = 2 T = 0.0(5% 6.0)	4095	18			
Modelled Rcomb6830			2851 (37.8%) 281 2747 (15.6%) 2727 2698 (14.8%) 2667	2861 (44.2%) 2806 2756 (21.5%) 2718 2704(29.8%) 2634	
RTD 6844.1	4155	35	2872 (14.8%) 2849 2810 (8.1%) 2796 2784 (25.0%) 2745 2728 (20.3%) 2696	2878 (19.6%) 2838 2816(75.4%) 2668 2643 (0.4%) 2640	L.E15AM08 B.TS12E15AM083 seeds in cooking installation
RTD 6844.2	4210	35			
RTD 6844.3	4160	35			
RTD 6844.4	4135	35			
Rcomb RTD 6844 X2-Test: df = 3 T = 2.5(5% 7.8)	4165	18			
Modelled Rcomb6844			2868 (19.1%) 2837 2814 (9.0%) 2798 2759 (19.5%) 2716 2708 (20.7%) 2668	2874 (91.9%) 2662 2652 (3.5%) 2636	

As each analyzed equid individual had an intact mandible, it was possible to sample all three molar teeth from each individual (Table 3). The total number of teeth per sample was determined by the size of the tooth. The third molar from EQ4 was sampled less due to breakage. Sequential sampling of teeth followed methods outlined by Bocherens et al. [82]. A series of 1.0 mm bands were drilled sequentially along the mesial lobe of each molar tooth. Dentin and cementum were removed from the equine teeth [83] prior to enamel sampling.

**Table 3:** Sample description from Area E at Tell eṣ-Ṣâfi/Gath.

Locus	Individual	Tooth	Total # of samples/tooth
19E82D04	EQ2	M1	20
19E82D04	EQ2	M2	26
19E82D04	EQ2	M3	19
19E83C09	EQ3	M1	14
19E83C09	EQ3	M2	16
19E83C09	EQ3	M3	16
20E93A05	EQ4	M1	19
20E93A05	EQ4	M2	24
20E93A05	EQ4	M3	6
20E93A12	EQ21	M1	31
20E93A12	EQ21	M2	26
20E93A12	EQ21	M3	17

Pretreatment of the samples followed the methods of Balasse and colleagues [84–86]. The tooth enamel was treated with a 2.5% NaOCl (sodium hypochlorite) solution overnight to remove organics and then rinsed five times with distilled water and treated with 0.1M CH_3_COOH (acetic acid, pH3) for four hours to remove diagenetic carbonates. The samples were then rinsed five times with distilled water and freeze-dried. Even-numbered samples were analyzed for strontium isotope ratios and odd-numbered samples were analyzed for carbon and oxygen isotope compositions. Strontium isotope measurements were performed on a Nu Plasma HR multi-collector inductively-coupled plasma mass spectrometer (MC-ICP-MS) at the University of Illinois, Urbana-Champaign Geology Department. Tooth enamel samples were dissolved in 0.5 ml of 3N nitric acid (HNO_3_) under clean lab conditions. Cation exchange columns loaded with Eichrom^®^ Sr spec resin and pre-conditioned with 3N HNO_3_ were prepared and the 0.5 ml samples were then loaded. Column blanks consisted of 0.5 ml of 3N HNO_3_. Columns were washed four times with 0.3 ml of 3N HNO_3_ and then strontium was eluted into 4 ml Teflon^®^ vials with 1 ml of 0.05N HNO_3_ and 3 ml of ultrapure deionized water (Milli-Q, Millipore) following Horwitz et al. [87]. Sample concentrations are measured and corrected to optimal range. Linear normalization of sample results was applied based on within-run trends in SRM 987 relative to its accepted value (0.710255). Analytical precision on repeated standard measurements was ± 0.00003.

Carbon and oxygen isotopic analyses were performed in the Department of Anthropology’s Stable Isotope Laboratory and the Mass Spectrometry Laboratory of the University of Illinois Urbana-Champaign. Approximately 700 μg of the prepared sample was weighed into individual vessels and reacted with 100% phosphoric acid (H_3_PO_4_) at 70°C in an automated Kiel III carbonate device in which CO_2_ is liberated from enamel, cryogenically distilled, and subsequently flowed to a Finnigan MAT 252 isotopic ratio mass spectrometer. Two laboratory standards (NBS18 and NBS 19) were interspersed, and replicates were run to ensure accuracy. Analytical precision is typically ±0.07‰ for δ^13^C and ±0.14‰ for δ^18^O.

## Supporting information

S1 Table Contextual and excavation data of *Equus asinus* (EQ2, EQ3, EQ4 and EQ21). (DOCX)

S2 Table Carbon and oxygen isotopic analysis of EQ2, EQ3, EQ4 and EQ21. (DOCX)

S3 Table Strontium isotopic analysis of EQ2, EQ3, EQ4 and EQ21. (DOCX)

S4 Table Statistical data: MDS PROC multidimensional scaling. (XLSX)

S1 Checklist Questionnaire on inclusivity in global research. (DOCX)
