# Supplementary material for: An isotopic perspective on equid selection in cult at Tell eṣ-Ṣâfi/Gath, Israel
Source: PLoS One. 2025 Jul 9;20(7):e0326421. doi: 10.1371/journal.pone.0326421 (PMC12240359; doi:10.1371/journal.pone.0326421)
Supplement: S1 Table — (DOCX) [file pone.0326421.s001.docx]

| **Individual** | **Locus** | **Basket** | **Tooth** | **Side** | **Distance from REJ (mm)** | **^13^C/^12^C vPDB** | **^18^O/^16^O vPDB** |
| --- | --- | --- | --- | --- | --- | --- | --- |
| EQ2 | 19E82D04 | 19E82D119 | LM1 | Right | 13.5 | -1.0 | 0.9 |
| EQ2 | 19E82D04 | 19E82D119 | LM1 | Right | 19.1 | -0.7 | 0.5 |
| EQ2 | 19E82D04 | 19E82D119 | LM1 | Right | 24.1 | -0.8 | 1.0 |
| EQ2 | 19E82D04 | 19E82D119 | LM1 | Right | 29.6 | -0.3 | 0.7 |
| EQ2 | 19E82D04 | 19E82D119 | LM1 | Right | 35.2 | 0.0 | 0.2 |
| EQ2 | 19E82D04 | 19E82D119 | LM1 | Right | 39.6 | 0.7 | 0.6 |
| EQ2 | 19E82D04 | 19E82D119 | LM1 | Right | 44.0 | 0.0 | 0.2 |
| EQ2 | 19E82D04 | 19E82D119 | LM1 | Right | 48.1 | 0.5 | 1.0 |
| EQ2 | 19E82D04 | 19E82D119 | LM1 | Right | 53.1 | -1.4 | 1.6 |
| EQ2 | 19E82D04 | 19E82D119 | LM1 | Right | 58.4 | -1.7 | 1.9 |
|  |  |  |  |  |  |  |  |
| EQ2 | 19E82D04 | 19E82D119 | LM2 | Right | 6.4 | -4.3 | -1.2 |
| EQ2 | 19E82D04 | 19E82D119 | LM2 | Right | 9.5 | -5.3 | 0.0 |
| EQ2 | 19E82D04 | 19E82D119 | LM2 | Right | 13.5 | -4.4 | -0.1 |
| EQ2 | 19E82D04 | 19E82D119 | LM2 | Right | 17.7 | -3.2 | 0.9 |
| EQ2 | 19E82D04 | 19E82D119 | LM2 | Right | 22.5 | -2.7 | 0.1 |
| EQ2 | 19E82D04 | 19E82D119 | LM2 | Right | 29.4 | -3.6 | 0.7 |
| EQ2 | 19E82D04 | 19E82D119 | LM2 | Right | 33.9 | -1.3 | -0.7 |
| EQ2 | 19E82D04 | 19E82D119 | LM2 | Right | 37.3 | -1.4 | 0.3 |
| EQ2 | 19E82D04 | 19E82D119 | LM2 | Right | 41.1 | -0.6 | 0.9 |
| EQ2 | 19E82D04 | 19E82D119 | LM2 | Right | 45.6 | -0.6 | 0.8 |
| EQ2 | 19E82D04 | 19E82D119 | LM2 | Right | 51.3 | -1.1 | 1.4 |
| EQ2 | 19E82D04 | 19E82D119 | LM2 | Right | 58.5 | 0.0 | 1.0 |
|  |  |  |  |  |  |  |  |
| EQ2 | 19E82D04 | 19E82D119 | LM3 | Right | 3.0 | -7.0 | 1.2 |
| EQ2 | 19E82D04 | 19E82D119 | LM3 | Right | 8.0 | -4.8 | 1.1 |
| EQ2 | 19E82D04 | 19E82D119 | LM3 | Right | 14.0 | -4.0 | 1.4 |
| EQ2 | 19E82D04 | 19E82D119 | LM3 | Right | 19.5 | -5.0 | 1.4 |
| EQ2 | 19E82D04 | 19E82D119 | LM3 | Right | 24.2 | -3.3 | 0.2 |
| EQ2 | 19E82D04 | 19E82D119 | LM3 | Right | 29.5 | -3.2 | 0.2 |
| EQ2 | 19E82D04 | 19E82D119 | LM3 | Right | 35.5 | -2.5 | 1.2 |
| EQ2 | 19E82D04 | 19E82D119 | LM3 | Right | 41.0 | -2.1 | 0.4 |
| EQ2 | 19E82D04 | 19E82D119 | LM3 | Right | 46.0 | -1.6 | 1.6 |
| EQ2 | 19E82D04 | 19E82D119 | LM3 | Right | 50.0 | -2.5 | 1.9 |
|  |  |  |  |  |  |  |  |
| EQ3 | 19E83C09 | 19E83C306 | LM1 | Right | 7.7 | -2.2 | 0.7 |
| EQ3 | 19E83C09 | 19E83C306 | LM1 | Right | 13.3 | 0.1 | 0.2 |
| EQ3 | 19E83C09 | 19E83C306 | LM1 | Right | 16.9 | 0.1 | -0.1 |
| EQ3 | 19E83C09 | 19E83C306 | LM1 | Right | 22.8 | -0.5 | 0.3 |
| EQ3 | 19E83C09 | 19E83C306 | LM1 | Right | 29.3 | -0.7 | 0.1 |
| EQ3 | 19E83C09 | 19E83C306 | LM1 | Right | 33.9 | 0.5 | 0.9 |
| EQ3 | 19E83C09 | 19E83C306 | LM1 | Right | 40.3 | -0.5 | 0.2 |
|  |  |  |  |  |  |  |  |
| EQ3 | 19E83C09 | 19E83C306 | LM2 | Right | 11.8 | -3.6 | 1.0 |
| EQ3 | 19E83C09 | 19E83C306 | LM2 | Right | 17.4 | -2.7 | 1.9 |
| EQ3 | 19E83C09 | 19E83C306 | LM2 | Right | 22.3 | -3.0 | 1.3 |
| EQ3 | 19E83C09 | 19E83C306 | LM2 | Right | 27.6 | -1.8 | 1.1 |
| EQ3 | 19E83C09 | 19E83C306 | LM2 | Right | 31.6 | -1.4 | 1.2 |
| EQ3 | 19E83C09 | 19E83C306 | LM2 | Right | 37.0 | 0.4 | 1.0 |
| EQ3 | 19E83C09 | 19E83C306 | LM2 | Right | 43.3 | 0.0 | -0.5 |
| EQ3 | 19E83C09 | 19E83C306 | LM2 | Right | 48.9 | 0.4 | 2.0 |
|  |  |  |  |  |  |  |  |
| EQ3 | 19E83C09 | 19E83C306 | LM3 | Right | 10.2 | -2.4 | 1.3 |
| EQ3 | 19E83C09 | 19E83C306 | LM3 | Right | 15.2 | -2.7 | 1.3 |
| EQ3 | 19E83C09 | 19E83C306 | LM3 | Right | 20.5 | -3.6 | 2.1 |
| EQ3 | 19E83C09 | 19E83C306 | LM3 | Right | 23.6 | -3.0 | 1.8 |
| EQ3 | 19E83C09 | 19E83C306 | LM3 | Right | 27.4 | -2.3 | 2.5 |
| EQ3 | 19E83C09 | 19E83C306 | LM3 | Right | 33.1 | -2.8 | 2.1 |
| EQ3 | 19E83C09 | 19E83C306 | LM3 | Right | 38.4 | -2.5 | 1.7 |
| EQ3 | 19E83C09 | 19E83C306 | LM3 | Right | 43.4 | -1.9 | 2.2 |
|  |  |  |  |  |  |  |  |
| EQ4 | 20E93A05 | 20E93A256 | LM1 | Left | 7.4 | -7.1 | 0.6 |
| EQ4 | 20E93A05 | 20E93A256 | LM1 | Left | 11.5 | -5.8 | 1.2 |
| EQ4 | 20E93A05 | 20E93A256 | LM1 | Left | 15.5 | -4.1 | 0.5 |
| EQ4 | 20E93A05 | 20E93A256 | LM1 | Left | 19.6 | -2.8 | 0.6 |
| EQ4 | 20E93A05 | 20E93A256 | LM1 | Left | 23.8 | -3.4 | 1.3 |
| EQ4 | 20E93A05 | 20E93A256 | LM1 | Left | 27.9 | -3.3 | 0.5 |
| EQ4 | 20E93A05 | 20E93A256 | LM1 | Left | 31.8 | -3.3 | -0.9 |
| EQ4 | 20E93A05 | 20E93A256 | LM1 | Left | 36.0 | -3.2 | -0.1 |
| EQ4 | 20E93A05 | 20E93A256 | LM1 | Left | 39.7 | -2.0 | -0.1 |
| EQ4 | 20E93A05 | 20E93A256 | LM1 | Left | 43.5 | -1.3 | 0.3 |
|  |  |  |  |  |  |  |  |
| EQ4 | 20E93A05 | 20E93A256 | LM2 | Left | 5.5 | -4.7 | 0.6 |
| EQ4 | 20E93A05 | 20E93A256 | LM2 | Left | 9.0 | -7.5 | 0.4 |
| EQ4 | 20E93A05 | 20E93A256 | LM2 | Left | 14.0 | -4.0 | 1.3 |
| EQ4 | 20E93A05 | 20E93A256 | LM2 | Left | 16.7 | -4.3 | 0.9 |
| EQ4 | 20E93A05 | 20E93A256 | LM2 | Left | 20.7 | -4.2 | 1.0 |
| EQ4 | 20E93A05 | 20E93A256 | LM2 | Left | 25.0 | -5.5 | 1.6 |
| EQ4 | 20E93A05 | 20E93A256 | LM2 | Left | 29.3 | -5.4 | 0.5 |
| EQ4 | 20E93A05 | 20E93A256 | LM2 | Left | 33.4 | -3.3 | 0.5 |
| EQ4 | 20E93A05 | 20E93A256 | LM2 | Left | 37.0 | -3.8 | 0.5 |
| EQ4 | 20E93A05 | 20E93A256 | LM2 | Left | 40.3 | -3.5 | 0.4 |
| EQ4 | 20E93A05 | 20E93A256 | LM2 | Left | 44.4 | -2.8 | 0.9 |
| EQ4 | 20E93A05 | 20E93A256 | LM2 | Left | 47.7 | -3.4 | 0.7 |
|  |  |  |  |  |  |  |  |
| EQ4 | 20E93A05 | 20E93A256 | LM3 | Left | 33.0 | -6.8 | 0.3 |
| EQ4 | 20E93A05 | 20E93A256 | LM3 | Left | 36.0 | -5.6 | 0.6 |
| EQ4 | 20E93A05 | 20E93A256 | LM3 | Left | 40.0 | -5.0 | 0.5 |
|  |  |  |  |  |  |  |  |
| EQ21 | 20E93A12 | 20E93A139 | LM1 | Right | 5.4 | -10.3 | -2.7 |
| EQ21 | 20E93A12 | 20E93A139 | LM1 | Right | 10.0 | -10.4 | -3.2 |
| EQ21 | 20E93A12 | 20E93A139 | LM1 | Right | 13.8 | -10.5 | -2.5 |
| EQ21 | 20E93A12 | 20E93A139 | LM1 | Right | 17.5 | -10.7 | -2.6 |
| EQ21 | 20E93A12 | 20E93A139 | LM1 | Right | 21.6 | -10.7 | -2.8 |
| EQ21 | 20E93A12 | 20E93A139 | LM1 | Right | 24.7 | -10.6 | -3.5 |
| EQ21 | 20E93A12 | 20E93A139 | LM1 | Right | 29.4 | -10.4 | -2.2 |
| EQ21 | 20E93A12 | 20E93A139 | LM1 | Right | 33.6 | -10.4 | -2.7 |
| EQ21 | 20E93A12 | 20E93A139 | LM1 | Right | 37.9 | -10.3 | -2.1 |
| EQ21 | 20E93A12 | 20E93A139 | LM1 | Right | 41.6 | -10.5 | -0.7 |
| EQ21 | 20E93A12 | 20E93A139 | LM1 | Right | 45.4 | -11.0 | -0.9 |
| EQ21 | 20E93A12 | 20E93A139 | LM1 | Right | 49.1 | -11.2 | -0.7 |
| EQ21 | 20E93A12 | 20E93A139 | LM1 | Right | 52.8 | -11.2 | -0.9 |
| EQ21 | 20E93A12 | 20E93A139 | LM1 | Right | 56.2 | -11.2 | -0.1 |
| EQ21 | 20E93A12 | 20E93A139 | LM1 | Right | 59.8 | -11.0 | -0.6 |
| EQ21 | 20E93A12 | 20E93A139 | LM1 | Right | 63.6 | -10.8 | -0.9 |
|  |  |  |  |  |  |  |  |
| EQ21 | 20E93A12 | 20E93A139 | LM2 | Right | 5.0 | -10.1 | -2.4 |
| EQ21 | 20E93A12 | 20E93A139 | LM2 | Right | 9.0 | -10.0 | -2.4 |
| EQ21 | 20E93A12 | 20E93A139 | LM2 | Right | 11.0 | -10.4 | -2.7 |
| EQ21 | 20E93A12 | 20E93A139 | LM2 | Right | 15.0 | -11.4 | -2.6 |
| EQ21 | 20E93A12 | 20E93A139 | LM2 | Right | 19.0 | -10.8 | -2.1 |
| EQ21 | 20E93A12 | 20E93A139 | LM2 | Right | 23.0 | -11.1 | -1.6 |
| EQ21 | 20E93A12 | 20E93A139 | LM2 | Right | 27.0 | -11.1 | -1.4 |
| EQ21 | 20E93A12 | 20E93A139 | LM2 | Right | 30.5 | -10.8 | -1.6 |
| EQ21 | 20E93A12 | 20E93A139 | LM2 | Right | 34.5 | -10.5 | -2.8 |
| EQ21 | 20E93A12 | 20E93A139 | LM2 | Right | 38.0 | -10.6 | -2.3 |
| EQ21 | 20E93A12 | 20E93A139 | LM2 | Right | 41.5 | -10.5 | -2.3 |
| EQ21 | 20E93A12 | 20E93A139 | LM2 | Right | 45.5 | -10.6 | -2.6 |
| EQ21 | 20E93A12 | 20E93A139 | LM2 | Right | 49.0 | -10.7 | -2.3 |
|  |  |  |  |  |  |  |  |
| EQ21 | 20E93A12 | 20E93A139 | LM3 | Right | 3.0 | -10.5 | -1.6 |
| EQ21 | 20E93A12 | 20E93A139 | LM3 | Right | 7.0 | -10.3 | -2.5 |
| EQ21 | 20E93A12 | 20E93A139 | LM3 | Right | 11.0 | -10.0 | -2.6 |
| EQ21 | 20E93A12 | 20E93A139 | LM3 | Right | 15.0 | -9.8 | -2.8 |
| EQ21 | 20E93A12 | 20E93A139 | LM3 | Right | 18.0 | -9.9 | -3.3 |
| EQ21 | 20E93A12 | 20E93A139 | LM3 | Right | 22.0 | -10.0 | -3.2 |
| EQ21 | 20E93A12 | 20E93A139 | LM3 | Right | 26.0 | -10.0 | -3.3 |
| EQ21 | 20E93A12 | 20E93A139 | LM3 | Right | 31.0 | -10.4 | -3.2 |
