# Supplementary material for: An isotopic perspective on equid selection in cult at Tell eṣ-Ṣâfi/Gath, Israel
Source: PLoS One. 2025 Jul 9;20(7):e0326421. doi: 10.1371/journal.pone.0326421 (PMC12240359; doi:10.1371/journal.pone.0326421)
Supplement: S2 Table — (DOCX) [file pone.0326421.s002.docx]

| **Individual** | **Locus** | **Basket** | **Tooth** | **Side** | **Distance from REJ (mm)** | **^87^Sr/^86^Sr** |
| --- | --- | --- | --- | --- | --- | --- |
| EQ2 | 19E82D04 | 19E82D119 | LM1 | Right | 9.6 | 0.708293 |
| EQ2 | 19E82D04 | 19E82D119 | LM1 | Right | 15.9 | 0.708271 |
| EQ2 | 19E82D04 | 19E82D119 | LM1 | Right | 21.1 | 0.708296 |
| EQ2 | 19E82D04 | 19E82D119 | LM1 | Right | 26.1 | 0.708292 |
| EQ2 | 19E82D04 | 19E82D119 | LM1 | Right | 31.8 | 0.708217 |
| EQ2 | 19E82D04 | 19E82D119 | LM1 | Right | 37.2 | 0.708262 |
| EQ2 | 19E82D04 | 19E82D119 | LM1 | Right | 40.1 | 0.708218 |
| EQ2 | 19E82D04 | 19E82D119 | LM1 | Right | 45.6 | 0.708214 |
| EQ2 | 19E82D04 | 19E82D119 | LM1 | Right | 50.8 | 0.708239 |
| EQ2 | 19E82D04 | 19E82D119 | LM1 | Right | 55.5 | 0.708225 |
|  |  |  |  |  |  |  |
| EQ2 | 19E82D04 | 19E82D119 | LM2 | Right | 4.8 | 0.708308 |
| EQ2 | 19E82D04 | 19E82D119 | LM2 | Right | 7.1 | 0.708219 |
| EQ2 | 19E82D04 | 19E82D119 | LM2 | Right | 12.3 | 0.708209 |
| EQ2 | 19E82D04 | 19E82D119 | LM2 | Right | 14.5 | 0.708194 |
| EQ2 | 19E82D04 | 19E82D119 | LM2 | Right | 19.9 | 0.708216 |
| EQ2 | 19E82D04 | 19E82D119 | LM2 | Right | 25.5 | 0.708204 |
| EQ2 | 19E82D04 | 19E82D119 | LM2 | Right | 31.2 | 0.708256 |
| EQ2 | 19E82D04 | 19E82D119 | LM2 | Right | 35.8 | 0.708244 |
| EQ2 | 19E82D04 | 19E82D119 | LM2 | Right | 39.3 | 0.708239 |
| EQ2 | 19E82D04 | 19E82D119 | LM2 | Right | 43.4 | 0.708248 |
| EQ2 | 19E82D04 | 19E82D119 | LM2 | Right | 46.9 | 0.708264 |
| EQ2 | 19E82D04 | 19E82D119 | LM2 | Right | 49.5 | 0.708265 |
| EQ2 | 19E82D04 | 19E82D119 | LM2 | Right | 54.3 | 0.708293 |
| EQ2 | 19E82D04 | 19E82D119 | LM2 | Right | 59.3 | 0.708277 |
|  |  |  |  |  |  |  |
| EQ2 | 19E82D04 | 19E82D119 | LM3 | Right | 6.0 | 0.708294 |
| EQ2 | 19E82D04 | 19E82D119 | LM3 | Right | 11.0 | 0.708240 |
| EQ2 | 19E82D04 | 19E82D119 | LM3 | Right | 17.0 | 0.708178 |
| EQ2 | 19E82D04 | 19E82D119 | LM3 | Right | 22.0 | 0.708260 |
| EQ2 | 19E82D04 | 19E82D119 | LM3 | Right | 26.4 | 0.708205 |
| EQ2 | 19E82D04 | 19E82D119 | LM3 | Right | 33.2 | 0.708181 |
| EQ2 | 19E82D04 | 19E82D119 | LM3 | Right | 39.0 | 0.708212 |
| EQ2 | 19E82D04 | 19E82D119 | LM3 | Right | 44.0 | 0.708200 |
| EQ2 | 19E82D04 | 19E82D119 | LM3 | Right | 48.8 | 0.708196 |
|  |  |  |  |  |  |  |
| EQ3 | 19E83C09 | 19E83C306 | LM1 | Right | 5.9 | 0.708231 |
| EQ3 | 19E83C09 | 19E83C306 | LM1 | Right | 10.2 | 0.708314 |
| EQ3 | 19E83C09 | 19E83C306 | LM1 | Right | 15.3 | 0.708303 |
| EQ3 | 19E83C09 | 19E83C306 | LM1 | Right | 19.7 | 0.708300 |
| EQ3 | 19E83C09 | 19E83C306 | LM1 | Right | 25.8 | 0.708332 |
| EQ3 | 19E83C09 | 19E83C306 | LM1 | Right | 31.8 | 0.708329 |
| EQ3 | 19E83C09 | 19E83C306 | LM1 | Right | 36.7 | 0.708304 |
|  |  |  |  |  |  |  |
| EQ3 | 19E83C09 | 19E83C306 | LM2 | Right | 9.2 | 0.708260 |
| EQ3 | 19E83C09 | 19E83C306 | LM2 | Right | 14.2 | 0.708248 |
| EQ3 | 19E83C09 | 19E83C306 | LM2 | Right | 19.8 | 0.708230 |
| EQ3 | 19E83C09 | 19E83C306 | LM2 | Right | 24.7 | 0.708225 |
| EQ3 | 19E83C09 | 19E83C306 | LM2 | Right | 29.6 | 0.708275 |
| EQ3 | 19E83C09 | 19E83C306 | LM2 | Right | 34.8 | 0.708291 |
| EQ3 | 19E83C09 | 19E83C306 | LM2 | Right | 39.8 | 0.708332 |
| EQ3 | 19E83C09 | 19E83C306 | LM2 | Right | 46.4 | 0.708327 |
|  |  |  |  |  |  |  |
| EQ3 | 19E83C09 | 19E83C306 | LM3 | Right | 8.5 | 0.708210 |
| EQ3 | 19E83C09 | 19E83C306 | LM3 | Right | 13.0 | 0.708134 |
| EQ3 | 19E83C09 | 19E83C306 | LM3 | Right | 18.0 | 0.708244 |
| EQ3 | 19E83C09 | 19E83C306 | LM3 | Right | 22.5 | 0.708246 |
| EQ3 | 19E83C09 | 19E83C306 | LM3 | Right | 25.7 | 0.708247 |
| EQ3 | 19E83C09 | 19E83C306 | LM3 | Right | 30.3 | 0.708269 |
| EQ3 | 19E83C09 | 19E83C306 | LM3 | Right | 35.6 | 0.708282 |
| EQ3 | 19E83C09 | 19E83C306 | LM3 | Right | 41.4 | 0.708243 |
|  |  |  |  |  |  |  |
| EQ4 | 20E93A05 | 20E93A256 | LM1 | Left | 9.1 | 0.708231 |
| EQ4 | 20E93A05 | 20E93A256 | LM1 | Left | 13.5 | 0.708234 |
| EQ4 | 20E93A05 | 20E93A256 | LM1 | Left | 17.4 | 0.708200 |
| EQ4 | 20E93A05 | 20E93A256 | LM1 | Left | 21.6 | 0.708219 |
| EQ4 | 20E93A05 | 20E93A256 | LM1 | Left | 26.1 | 0.708215 |
| EQ4 | 20E93A05 | 20E93A256 | LM1 | Left | 30.0 | 0.708300 |
| EQ4 | 20E93A05 | 20E93A256 | LM1 | Left | 34.1 | 0.708304 |
| EQ4 | 20E93A05 | 20E93A256 | LM1 | Left | 38.1 | 0.708308 |
| EQ4 | 20E93A05 | 20E93A256 | LM1 | Left | 41.8 | 0.708302 |
|  |  |  |  |  |  |  |
| EQ4 | 20E93A05 | 20E93A256 | LM2 | Left | 3.5 | 0.708280 |
| EQ4 | 20E93A05 | 20E93A256 | LM2 | Left | 7.2 | 0.708278 |
| EQ4 | 20E93A05 | 20E93A256 | LM2 | Left | 11.2 | 0.708229 |
| EQ4 | 20E93A05 | 20E93A256 | LM2 | Left | 15.2 | 0.708210 |
| EQ4 | 20E93A05 | 20E93A256 | LM2 | Left | 19.0 | 0.708226 |
| EQ4 | 20E93A05 | 20E93A256 | LM2 | Left | 23.2 | 0.708205 |
| EQ4 | 20E93A05 | 20E93A256 | LM2 | Left | 27.6 | 0.708224 |
| EQ4 | 20E93A05 | 20E93A256 | LM2 | Left | 30.7 | 0.708199 |
| EQ4 | 20E93A05 | 20E93A256 | LM2 | Left | 34.6 | 0.708217 |
| EQ4 | 20E93A05 | 20E93A256 | LM2 | Left | 38.5 | 0.708204 |
| EQ4 | 20E93A05 | 20E93A256 | LM2 | Left | 42.2 | 0.708222 |
| EQ4 | 20E93A05 | 20E93A256 | LM2 | Left | 46.2 | 0.708226 |
|  |  |  |  |  |  |  |
| EQ4 | 20E93A05 | 20E93A256 | LM3 | Left | 31.0 | 0.708282 |
| EQ4 | 20E93A05 | 20E93A256 | LM3 | Left | 34.0 | 0.708272 |
| EQ4 | 20E93A05 | 20E93A256 | LM3 | Left | 38.0 | 0.708222 |
|  |  |  |  |  |  |  |
| EQ21 | 20E93A12 | 20E93A139 | LM1 | Right | 7.3 | 0.708390 |
| EQ21 | 20E93A12 | 20E93A139 | LM1 | Right | 12.1 | 0.708356 |
| EQ21 | 20E93A12 | 20E93A139 | LM1 | Right | 15.6 | 0.708398 |
| EQ21 | 20E93A12 | 20E93A139 | LM1 | Right | 19.3 | 0.708392 |
| EQ21 | 20E93A12 | 20E93A139 | LM1 | Right | 23.6 | 0.708388 |
| EQ21 | 20E93A12 | 20E93A139 | LM1 | Right | 27.0 | 0.708400 |
| EQ21 | 20E93A12 | 20E93A139 | LM1 | Right | 31.9 | 0.708415 |
| EQ21 | 20E93A12 | 20E93A139 | LM1 | Right | 35.4 | 0.708406 |
| EQ21 | 20E93A12 | 20E93A139 | LM1 | Right | 39.7 | 0.708384 |
| EQ21 | 20E93A12 | 20E93A139 | LM1 | Right | 43.6 | 0.708385 |
| EQ21 | 20E93A12 | 20E93A139 | LM1 | Right | 47.1 | 0.708399 |
| EQ21 | 20E93A12 | 20E93A139 | LM1 | Right | 50.1 | 0.708414 |
| EQ21 | 20E93A12 | 20E93A139 | LM1 | Right | 55.0 | 0.708399 |
| EQ21 | 20E93A12 | 20E93A139 | LM1 | Right | 57.6 | 0.708388 |
| EQ21 | 20E93A12 | 20E93A139 | LM1 | Right | 61.6 | 0.708398 |
|  |  |  |  |  |  |  |
| EQ21 | 20E93A12 | 20E93A139 | LM2 | Right | 2.5 | 0.708416 |
| EQ21 | 20E93A12 | 20E93A139 | LM2 | Right | 7.0 | 0.708414 |
| EQ21 | 20E93A12 | 20E93A139 | LM2 | Right | 10.0 | 0.708412 |
| EQ21 | 20E93A12 | 20E93A139 | LM2 | Right | 13.0 | 0.708373 |
| EQ21 | 20E93A12 | 20E93A139 | LM2 | Right | 17.0 | 0.708392 |
| EQ21 | 20E93A12 | 20E93A139 | LM2 | Right | 21.0 | 0.708398 |
| EQ21 | 20E93A12 | 20E93A139 | LM2 | Right | 25.5 | 0.708404 |
| EQ21 | 20E93A12 | 20E93A139 | LM2 | Right | 29.0 | 0.708393 |
| EQ21 | 20E93A12 | 20E93A139 | LM2 | Right | 32.5 | 0.708398 |
| EQ21 | 20E93A12 | 20E93A139 | LM2 | Right | 36.0 | 0.708395 |
| EQ21 | 20E93A12 | 20E93A139 | LM2 | Right | 40.0 | 0.708389 |
| EQ21 | 20E93A12 | 20E93A139 | LM2 | Right | 44.0 | 0.708391 |
| EQ21 | 20E93A12 | 20E93A139 | LM2 | Right | 47.0 | 0.708395 |
|  |  |  |  |  |  |  |
| EQ21 | 20E93A12 | 20E93A139 | LM3 | Right | 1.5 | 0.708439 |
| EQ21 | 20E93A12 | 20E93A139 | LM3 | Right | 5.0 | 0.708406 |
| EQ21 | 20E93A12 | 20E93A139 | LM3 | Right | 9.0 | 0.708405 |
| EQ21 | 20E93A12 | 20E93A139 | LM3 | Right | 13.0 | 0.708402 |
| EQ21 | 20E93A12 | 20E93A139 | LM3 | Right | 16.0 | 0.708375 |
| EQ21 | 20E93A12 | 20E93A139 | LM3 | Right | 20.0 | 0.708384 |
| EQ21 | 20E93A12 | 20E93A139 | LM3 | Right | 24.0 | 0.708386 |
| EQ21 | 20E93A12 | 20E93A139 | LM3 | Right | 28.0 | 0.708390 |
| EQ21 | 20E93A12 | 20E93A139 | LM3 | Right | 32.5 | 0.708391 |
