# Supplementary material for: An isotopic perspective on equid selection in cult at Tell eṣ-Ṣâfi/Gath, Israel
Source: PLoS One. 2025 Jul 9;20(7):e0326421. doi: 10.1371/journal.pone.0326421 (PMC12240359; doi:10.1371/journal.pone.0326421)
Supplement: S3 Table — (DOCX) [file pone.0326421.s003.docx]

| Obs | _LABEL_ | _NAME_ | Dim1 | Dim2 | Dim3 | Dim4 |
| --- | --- | --- | --- | --- | --- | --- |
| 1 | EQ1 | EQ1 | 10.7835234 | 0.690411 | 0.003373 | 0.001697 |
| 2 | EQ1 | EQ12 | 2.96492618 | -0.75524 | -0.00101 | 7.88E-06 |
| 3 | EQ1 | EQ13 | 3.54499147 | -0.44753 | -0.00094 | -0.00028 |
| 4 | EQ1 | EQ14 | 3.54122593 | -0.39334 | -0.00095 | -0.00031 |
| 5 | EQ1 | EQ15 | 4.6162159 | -1.43787 | -0.00067 | 0.000537 |
| 6 | EQ1 | EQ16 | 2.11933778 | -1.56955 | -0.00119 | 0.000398 |
| 7 | EQ1 | EQ17 | 2.69267897 | -1.16506 | -0.00106 | 0.00023 |
| 8 | EQ1 | EQ18 | 2.76453311 | -0.75653 | -0.00101 | 3.18E-05 |
| 9 | EQ1 | EQ19 | 3.0487798 | -0.5194 | -0.001 | -9.6E-05 |
| 10 | EQ1 | EQ110 | 4.45688031 | -0.5874 | -0.00067 | 0.000109 |
| 11 | EQ1 | EQ111 | 3.78996589 | -1.08785 | -0.00089 | 0.000172 |
| 12 | EQ1 | EQ112 | 3.3655106 | -0.74977 | -0.00096 | -5E-05 |
| 13 | EQ1 | EQ113 | 3.45995797 | -0.66639 | -0.00095 | -0.00012 |
| 14 | EQ1 | EQ114 | 3.62303183 | -0.12802 | -0.00095 | -0.0005 |
| 15 | EQ1 | EQ115 | 4.48061663 | -0.92901 | -0.00069 | 0.000296 |
| 16 | EQ1 | EQ116 | 1.62909292 | 1.157996 | -0.00017 | -0.00064 |
| 17 | EQ1 | EQ117 | 2.3850354 | 0.377176 | -0.00065 | -0.00031 |
| 18 | EQ1 | EQ118 | 1.96383498 | -0.77424 | -0.001 | 7.61E-05 |
| 19 | EQ1 | EQ119 | 3.13848981 | -0.36784 | -0.00099 | -0.00013 |
| 20 | EQ1 | EQ120 | 3.77892473 | 0.513698 | -0.00095 | -0.00047 |
| 21 | EQ1 | EQ121 | 3.9652735 | 0.717104 | -0.00072 | -0.00031 |
| 22 | EQ1 | EQ122 | 4.39897008 | 0.246034 | -0.00069 | -0.00023 |
| 23 | EQ1 | EQ123 | -3.30487261 | 2.919436 | 0.000694 | -2.1E-05 |
| 24 | EQ1 | EQ124 | -3.69375698 | 2.745582 | 0.000533 | 0.000145 |
| 25 | EQ1 | EQ125 | -0.49688145 | 2.901591 | 0.001131 | -4.3E-05 |
| 26 | EQ2_EQ4 | EQ2_EQ4 | 5.785743 | 0.383986 | 0.000344 | 0.000382 |
| 27 | EQ2_EQ4 | EQ2_EQ42 | 6.09300629 | 0.75151 | 0.000771 | 0.000299 |
| 28 | EQ2_EQ4 | EQ2_EQ43 | 6.02689082 | 0.318088 | 0.000572 | 0.000337 |
| 29 | EQ2_EQ4 | EQ2_EQ44 | 6.46729278 | 0.616098 | 0.001113 | 0.000216 |
| 30 | EQ2_EQ4 | EQ2_EQ45 | 6.70357244 | 1.182894 | 0.00152 | 0.000184 |
| 31 | EQ2_EQ4 | EQ2_EQ46 | 7.51708841 | 0.78531 | 0.002063 | 0.000212 |
| 32 | EQ2_EQ4 | EQ2_EQ47 | 6.74175 | 1.181657 | 0.001553 | 0.000181 |
| 33 | EQ2_EQ4 | EQ2_EQ48 | 7.3082386 | 0.357528 | 0.00187 | 7.9E-05 |
| 34 | EQ2_EQ4 | EQ2_EQ49 | 5.47595338 | -0.33669 | -9.4E-05 | 0.000457 |
| 35 | EQ2_EQ4 | EQ2_EQ410 | 5.19422406 | -0.69661 | -0.00034 | 0.000473 |
| 36 | EQ2_EQ4 | EQ2_EQ411 | 2.40199453 | 2.22494 | 0.000389 | -0.0017 |
| 37 | EQ2_EQ4 | EQ2_EQ412 | 1.44016403 | 0.948444 | -0.00027 | -0.00063 |
| 38 | EQ2_EQ4 | EQ2_EQ413 | 2.33756369 | 1.146935 | -0.00033 | -0.00057 |
| 39 | EQ2_EQ4 | EQ2_EQ414 | 3.59533918 | 0.256097 | -0.00123 | -0.00046 |
| 40 | EQ2_EQ4 | EQ2_EQ415 | 4.05262856 | 1.090101 | -0.00047 | -0.00017 |
| 41 | EQ2_EQ4 | EQ2_EQ416 | 3.13933905 | 0.355686 | -0.00156 | -0.00036 |
| 42 | EQ2_EQ4 | EQ2_EQ417 | 5.38975101 | 1.957039 | 0.00077 | 0.000185 |
| 43 | EQ2_EQ4 | EQ2_EQ418 | 5.3633534 | 0.980862 | 0.00019 | 0.000333 |
| 44 | EQ2_EQ4 | EQ2_EQ419 | 6.16071504 | 0.397399 | 0.000733 | 0.000296 |
| 45 | EQ2_EQ4 | EQ2_EQ420 | 6.18126213 | 0.491205 | 0.000781 | 0.000289 |
| 46 | EQ2_EQ4 | EQ2_EQ421 | 5.72260822 | -0.12119 | 0.000164 | 0.000423 |
| 47 | EQ2_EQ4 | EQ2_EQ422 | 6.83915645 | 0.342438 | 0.001433 | 0.000121 |
| 48 | EQ2_EQ4 | EQ2_EQ423 | 6.83915647 | 0.342438 | 0.001403 | 0.000138 |
| 49 | EQ2_EQ4 | EQ2_EQ424 | 6.83915645 | 0.342438 | 0.001436 | 0.000133 |
| 50 | EQ2_EQ4 | EQ2_EQ425 | -0.18253271 | -0.29521 | -0.00101 | 0.00025 |
| 51 | EQ2_EQ4 | EQ2_EQ426 | 2.04096032 | -0.12418 | -0.00075 | -0.00021 |
| 52 | EQ2_EQ4 | EQ2_EQ427 | 2.84551116 | -0.36388 | -0.00118 | -0.0001 |
| 53 | EQ2_EQ4 | EQ2_EQ428 | 1.78890192 | -0.40619 | -0.00084 | -0.00011 |
| 54 | EQ2_EQ4 | EQ2_EQ429 | 3.45775783 | 0.923327 | -0.00099 | -0.00033 |
| 55 | EQ2_EQ4 | EQ2_EQ430 | 3.57328814 | 0.919686 | -0.00092 | -0.00032 |
| 56 | EQ2_EQ4 | EQ2_EQ431 | 4.34431613 | -0.02053 | -0.00069 | -0.00032 |
| 57 | EQ2_EQ4 | EQ2_EQ432 | 4.64575668 | 0.834732 | -0.00032 | 0.000125 |
| 58 | EQ2_EQ4 | EQ2_EQ433 | 5.26973132 | -0.3695 | -0.00026 | 0.00044 |
| 59 | EQ2_EQ4 | EQ2_EQ434 | 4.34296276 | -0.69353 | -0.00072 | 0.000103 |
| 60 | EQ21_and_Caprines | EQ21_and_Caprines | -3.70473532 | 3.278666 | 0.001003 | -0.00018 |
| 61 | EQ21_and_Caprines | EQ21_and_Caprines2 | -3.83277952 | 3.823065 | 0.001451 | -0.0005 |
| 62 | EQ21_and_Caprines | EQ21_and_Caprines3 | -3.94019187 | 3.118391 | 0.000787 | -0.00012 |
| 63 | EQ21_and_Caprines | EQ21_and_Caprines4 | -4.11645098 | 3.216997 | 0.000828 | -0.00018 |
| 64 | EQ21_and_Caprines | EQ21_and_Caprines5 | -4.11915064 | 3.356835 | 0.000949 | -0.00025 |
| 65 | EQ21_and_Caprines | EQ21_and_Caprines6 | -4.05307618 | 4.108229 | 0.001649 | -0.00057 |
| 66 | EQ21_and_Caprines | EQ21_and_Caprines7 | -3.79463648 | 2.812475 | 0.000552 | 7.59E-05 |
| 67 | EQ21_and_Caprines | EQ21_and_Caprines8 | -3.85067521 | 3.301588 | 0.000977 | -0.00021 |
| 68 | EQ21_and_Caprines | EQ21_and_Caprines9 | -3.70504467 | 2.720488 | 0.000463 | 0.000147 |
| 69 | EQ21_and_Caprines | EQ21_and_Caprines10 | -3.83288788 | 1.328853 | -0.00063 | 0.001021 |
| 70 | EQ21_and_Caprines | EQ21_and_Caprines11 | -4.26137008 | 1.450775 | -0.00071 | 0.000698 |
| 71 | EQ21_and_Caprines | EQ21_and_Caprines12 | -4.51229554 | 1.253446 | -0.00084 | 0.000623 |
| 72 | EQ21_and_Caprines | EQ21_and_Caprines13 | -4.50179052 | 1.434069 | -0.0007 | 0.000632 |
| 73 | EQ21_and_Caprines | EQ21_and_Caprines14 | -4.47205198 | 0.63099 | -0.00121 | 0.000834 |
| 74 | EQ21_and_Caprines | EQ21_and_Caprines15 | -4.31071517 | 1.179936 | -0.00093 | 0.000633 |
| 75 | EQ21_and_Caprines | EQ21_and_Caprines16 | -4.10593592 | 1.449906 | -0.00071 | 0.000751 |
| 76 | EQ21_and_Caprines | EQ21_and_Caprines17 | -3.5368394 | 3.069598 | 0.000846 | -5.4E-05 |
| 77 | EQ21_and_Caprines | EQ21_and_Caprines18 | -3.38310946 | 3.035549 | 0.000841 | -2.3E-05 |
| 78 | EQ21_and_Caprines | EQ21_and_Caprines19 | -3.81712629 | 3.352539 | 0.001034 | -0.00023 |
| 79 | EQ21_and_Caprines | EQ21_and_Caprines20 | -4.84554131 | 3.178572 | 0.000681 | -0.00015 |
| 80 | EQ21_and_Caprines | EQ21_and_Caprines21 | -4.16912803 | 2.691159 | 0.000373 | 0.000129 |
| 81 | EQ21_and_Caprines | EQ21_and_Caprines22 | -4.43374384 | 2.128229 | -0.00012 | 0.000451 |
| 82 | EQ21_and_Caprines | EQ21_and_Caprines23 | -4.40868261 | 1.969524 | -0.00025 | 0.000536 |
| 83 | EQ21_and_Caprines | EQ21_and_Caprines24 | -4.20245835 | 2.175416 | -6.7E-05 | 0.000464 |
| 84 | EQ21_and_Caprines | EQ21_and_Caprines25 | -3.93762147 | 3.413206 | 0.001051 | -0.00027 |
| 85 | EQ21_and_Caprines | EQ21_and_Caprines26 | -4.00914041 | 2.913286 | 0.000594 | -1.8E-06 |
| 86 | EQ21_and_Caprines | EQ21_and_Caprines27 | -3.88383587 | 2.913241 | 0.000623 | 6.37E-06 |
| 87 | EQ21_and_Caprines | EQ21_and_Caprines28 | -4.05608046 | 3.156041 | 0.00079 | -0.00015 |
| 88 | EQ21_and_Caprines | EQ21_and_Caprines29 | -4.13164286 | 2.901857 | 0.000556 | -2.9E-06 |
| 89 | EQ21_and_Caprines | EQ21_and_Caprines30 | -3.81736361 | 2.259545 | 2.49E-05 | 0.000407 |
| 90 | EQ21_and_Caprines | EQ21_and_Caprines31 | -3.70619466 | 3.097699 | 0.00083 | -9.1E-05 |
| 91 | EQ21_and_Caprines | EQ21_and_Caprines32 | -3.38882395 | 3.20435 | 0.001025 | -8.8E-05 |
| 92 | EQ21_and_Caprines | EQ21_and_Caprines33 | -3.24850715 | 3.493178 | 0.001368 | -0.00015 |
| 93 | EQ21_and_Caprines | EQ21_and_Caprines34 | -3.42026688 | 3.97425 | 0.001755 | -0.00039 |
| 94 | EQ21_and_Caprines | EQ21_and_Caprines35 | -3.4327439 | 3.850861 | 0.001637 | -0.00035 |
| 95 | EQ21_and_Caprines | EQ21_and_Caprines36 | -3.50606005 | 3.968289 | 0.001717 | -0.00041 |
| 96 | EQ21_and_Caprines | EQ21_and_Caprines37 | -3.85583656 | 3.794235 | 0.001446 | -0.00038 |
| 97 | EQ21_and_Caprines | EQ21_and_Caprines38 | -3.85583653 | 3.794235 | 0.001437 | -0.00038 |
| 98 | EQ2_EQ4 | EQ2_EQ435 | 4.60514321 | 0.539218 | -0.00047 | 5.89E-05 |
| 99 | EQ2_EQ4 | EQ2_EQ436 | 6.87124659 | 1.178986 | 0.001667 | 0.000173 |
| 100 | EQ2_EQ4 | EQ2_EQ437 | 6.79445808 | 1.490653 | 0.001727 | 0.000165 |
| 101 | EQ2_EQ4 | EQ2_EQ438 | 6.27083919 | 1.034193 | 0.001055 | 0.000251 |
| 102 | EQ2_EQ4 | EQ2_EQ439 | 6.05354536 | 1.218437 | 0.000923 | 0.000272 |
| 103 | EQ2_EQ4 | EQ2_EQ440 | 7.29092229 | 0.476903 | 0.001864 | 0.000121 |
| 104 | EQ2_EQ4 | EQ2_EQ441 | 6.2169029 | 1.117961 | 0.001036 | 0.000255 |
| 105 | EQ2_EQ4 | EQ2_EQ442 | 3.16318893 | 0.127857 | -0.00134 | -0.00036 |
| 106 | EQ2_EQ4 | EQ2_EQ443 | 4.18766316 | -0.69459 | -0.00076 | 1.83E-05 |
| 107 | EQ2_EQ4 | EQ2_EQ444 | 3.78544806 | -0.21495 | -0.00084 | -0.00054 |
| 108 | EQ2_EQ4 | EQ2_EQ445 | 5.02595423 | 0.0949 | -0.00034 | 0.000306 |
| 109 | EQ2_EQ4 | EQ2_EQ446 | 5.40640704 | 0.072715 | -7.6E-05 | 0.000412 |
| 110 | EQ2_EQ4 | EQ2_EQ447 | 7.21793923 | 0.329861 | 0.001787 | 7.79E-05 |
| 111 | EQ2_EQ4 | EQ2_EQ448 | 6.74115219 | 1.796172 | 0.001824 | 0.000152 |
| 112 | EQ2_EQ4 | EQ2_EQ449 | 7.30441512 | -0.655 | 0.001634 | -0.00012 |
| 113 | EQ2_EQ4 | EQ2_EQ450 | 4.42534399 | -0.13353 | -0.00066 | -0.00016 |
| 114 | EQ2_EQ4 | EQ2_EQ451 | 4.10366985 | -0.17825 | -0.00072 | -0.00054 |
| 115 | EQ2_EQ4 | EQ2_EQ452 | 3.24324091 | -0.98095 | -0.00099 | 9.5E-05 |
| 116 | EQ2_EQ4 | EQ2_EQ453 | 3.87367979 | -0.61917 | -0.00083 | -0.00015 |
| 117 | EQ2_EQ4 | EQ2_EQ454 | 4.58941745 | -1.26859 | -0.00067 | 0.000471 |
| 118 | EQ2_EQ4 | EQ2_EQ455 | 4.11609111 | -0.9485 | -0.0008 | 0.000163 |
| 119 | EQ2_EQ4 | EQ2_EQ456 | 4.37067375 | -0.48642 | -0.00069 | -1.3E-05 |
| 120 | EQ2_EQ4 | EQ2_EQ457 | 4.96581691 | -0.94391 | -0.00048 | 0.000471 |
| 121 | EQ2_EQ4 | EQ2_EQ458 | -0.33509122 | 0.212481 | -0.00088 | -9.7E-05 |
| 122 | EQ2_EQ4 | EQ2_EQ459 | 1.00813578 | -0.22025 | -0.00063 | -0.00015 |
| 123 | EQ2_EQ4 | EQ2_EQ460 | 2.6726246 | 0.551771 | -0.00089 | -0.00034 |
| 124 | EQ2_EQ4 | EQ2_EQ461 | 4.0226383 | 0.555142 | -0.00066 | -0.00043 |
| 125 | EQ2_EQ4 | EQ2_EQ462 | 3.39419021 | -0.15267 | -0.00107 | -0.00036 |
| 126 | EQ2_EQ4 | EQ2_EQ463 | 3.43011308 | 0.628715 | -0.00176 | -0.00015 |
| 127 | EQ2_EQ4 | EQ2_EQ464 | 3.34033542 | 2.021758 | 9.87E-05 | -0.00106 |
| 128 | EQ2_EQ4 | EQ2_EQ465 | 3.53781554 | 1.213803 | -0.00059 | -0.00034 |
| 129 | EQ2_EQ4 | EQ2_EQ466 | 4.73003055 | 1.266499 | -6.5E-05 | 0.000147 |
| 130 | EQ2_EQ4 | EQ2_EQ467 | 5.44219647 | 0.942582 | 0.000238 | 0.000342 |
| 131 | EQ2_EQ4 | EQ2_EQ468 | 2.02947824 | 0.459435 | -0.00052 | -0.00049 |
| 132 | EQ2_EQ4 | EQ2_EQ469 | -0.73289355 | 0.397795 | -0.00131 | 0.00035 |
| 133 | EQ2_EQ4 | EQ2_EQ470 | 2.79677641 | -0.21071 | -0.00043 | 0.000282 |
| 134 | EQ2_EQ4 | EQ2_EQ471 | 2.50445233 | 0.158905 | -0.00021 | -0.00016 |
| 135 | EQ2_EQ4 | EQ2_EQ472 | 2.57087556 | 0.097398 | -0.00021 | -0.00014 |
| 136 | EQ2_EQ4 | EQ2_EQ473 | 1.3990186 | -0.66665 | -0.00021 | -0.00017 |
| 137 | EQ2_EQ4 | EQ2_EQ474 | 1.33311282 | 0.469393 | -0.00035 | -0.00033 |
| 138 | EQ2_EQ4 | EQ2_EQ475 | 3.42455265 | 0.593321 | -0.00094 | -6.1E-05 |
| 139 | EQ2_EQ4 | EQ2_EQ476 | 2.94786023 | 0.615626 | -0.00036 | -0.00037 |
| 140 | EQ2_EQ4 | EQ2_EQ477 | 3.25872949 | 0.700432 | -0.00042 | -0.00038 |
| 141 | EQ2_EQ4 | EQ2_EQ478 | 4.03385358 | 0.235029 | 0.000254 | -0.00102 |
| 142 | EQ2_EQ4 | EQ2_EQ479 | 3.3701867 | 0.365893 | -0.00037 | -0.00024 |
| 143 | EQ2_EQ4 | EQ2_EQ480 | -0.03960836 | 0.590857 | -0.00081 | 0.000186 |
| 144 | EQ2_EQ4 | EQ2_EQ481 | 1.16207444 | 0.348601 | -0.00043 | -0.00024 |
| 145 | EQ2_EQ4 | EQ2_EQ482 | 1.75531933 | 0.466641 | -0.00024 | -0.00028 |
| 146 | EQ21_and_Caprines | EQ21_and_Caprines39 | -2.4427865 | -2.27428 | 5.94E-05 | -0.00035 |
| 147 | EQ21_and_Caprines | EQ21_and_Caprines40 | -4.65898283 | -2.11752 | 0.000329 | 0.000108 |
| 148 | EQ21_and_Caprines | EQ21_and_Caprines41 | -4.13037918 | -2.51183 | 0.000376 | 0.000304 |
| 149 | EQ21_and_Caprines | EQ21_and_Caprines42 | -2.33337961 | -0.96354 | -0.00017 | 0.00015 |
| 150 | EQ21_and_Caprines | EQ21_and_Caprines43 | -1.38846593 | -0.13605 | -0.00065 | 0.000124 |
| 151 | EQ21_and_Caprines | EQ21_and_Caprines44 | -0.60078771 | 0.069041 | -0.00099 | -0.00014 |
| 152 | EQ21_and_Caprines | EQ21_and_Caprines45 | -0.60078831 | 0.069041 | -0.00073 | -0.00028 |
| 153 | EQ21_and_Caprines | EQ21_and_Caprines46 | -1.12919735 | -3.04508 | 0.000168 | -0.00021 |
| 154 | EQ21_and_Caprines | EQ21_and_Caprines47 | -1.59670906 | -2.37588 | 3.58E-05 | -6.3E-05 |
| 155 | EQ21_and_Caprines | EQ21_and_Caprines48 | -2.99679867 | -1.24019 | -2.9E-05 | 0.000141 |
| 156 | EQ21_and_Caprines | EQ21_and_Caprines49 | -3.50634558 | 1.491057 | -0.00048 | 0.000313 |
| 157 | EQ21_and_Caprines | EQ21_and_Caprines50 | -3.09732237 | 0.567187 | -0.00061 | 0.000207 |
| 158 | EQ21_and_Caprines | EQ21_and_Caprines51 | -2.47017599 | -1.99551 | 0.000179 | 0.000248 |
| 159 | EQ21_and_Caprines | EQ21_and_Caprines52 | -1.93739597 | -0.08398 | -0.00046 | 0.000281 |
| 160 | EQ21_and_Caprines | EQ21_and_Caprines53 | -1.63891463 | 1.881441 | -0.00077 | -0.00017 |
| 161 | EQ21_and_Caprines | EQ21_and_Caprines54 | -2.88269056 | 2.426537 | -0.0007 | -0.00012 |
| 162 | EQ21_and_Caprines | EQ21_and_Caprines55 | -2.92690768 | 2.182886 | -0.00061 | -5.5E-05 |
| 163 | EQ21_and_Caprines | EQ21_and_Caprines56 | -2.89295124 | 0.511218 | -0.00056 | 0.000315 |
| 164 | EQ21_and_Caprines | EQ21_and_Caprines57 | -2.67852877 | -0.75697 | -0.00023 | 0.00021 |
| 165 | EQ21_and_Caprines | EQ21_and_Caprines58 | -3.75024432 | -2.38517 | 0.000351 | -8.8E-05 |
| 166 | EQ21_and_Caprines | EQ21_and_Caprines59 | -4.74615254 | -3.05582 | 0.000639 | -7.9E-05 |
| 167 | EQ21_and_Caprines | EQ21_and_Caprines60 | -3.90897026 | -1.05297 | 8.2E-05 | 9.6E-05 |
| 168 | EQ21_and_Caprines | EQ21_and_Caprines61 | -3.5097123 | -0.98513 | -1.8E-05 | 0.000139 |
| 169 | EQ21_and_Caprines | EQ21_and_Caprines62 | -3.19770016 | 1.492455 | -0.00036 | 0.000137 |
| 170 | EQ21_and_Caprines | EQ21_and_Caprines63 | -2.73170205 | 1.955872 | -0.00073 | -3.5E-05 |
| 171 | EQ21_and_Caprines | EQ21_and_Caprines64 | -2.44288666 | 2.386929 | -0.00117 | 0.000227 |
| 172 | EQ21_and_Caprines | EQ21_and_Caprines65 | -2.54206064 | 1.578108 | -0.00064 | 2.14E-05 |
| 173 | EQ21_and_Caprines | EQ21_and_Caprines66 | -2.54206061 | 1.578108 | -0.00065 | 2.53E-05 |
| 174 | EQ21_and_Caprines | EQ21_and_Caprines67 | 1.33197605 | -2.08216 | 0.000183 | -0.00057 |
| 175 | EQ21_and_Caprines | EQ21_and_Caprines68 | 2.89501839 | -0.00883 | -0.00022 | -8.5E-05 |
| 176 | EQ21_and_Caprines | EQ21_and_Caprines69 | 2.76840686 | 2.087438 | -0.0004 | -0.00091 |
| 177 | EQ21_and_Caprines | EQ21_and_Caprines70 | 2.56745502 | 2.584705 | -0.00034 | -0.00087 |
| 178 | EQ21_and_Caprines | EQ21_and_Caprines71 | -3.49150796 | 0.469628 | -0.00043 | 0.00027 |
| 179 | EQ21_and_Caprines | EQ21_and_Caprines72 | -3.02743035 | 1.85513 | -0.00045 | 2.9E-05 |
| 180 | EQ21_and_Caprines | EQ21_and_Caprines73 | -2.61136322 | 2.214836 | -0.00087 | -6.6E-05 |
| 181 | EQ21_and_Caprines | EQ21_and_Caprines74 | -2.89758328 | 0.621162 | -0.00054 | 0.000313 |
| 182 | EQ21_and_Caprines | EQ21_and_Caprines75 | -2.83208153 | -4.11569 | 0.000468 | -0.00013 |
| 183 | EQ21_and_Caprines | EQ21_and_Caprines76 | -2.49924943 | -5.38568 | 0.000647 | -0.00022 |
| 184 | EQ21_and_Caprines | EQ21_and_Caprines77 | -2.49924943 | -5.38568 | 0.000654 | -0.00022 |
| 185 | EQ21_and_Caprines | EQ21_and_Caprines78 | -6.83312047 | -3.20083 | 0.001028 | -2.8E-05 |
| 186 | EQ21_and_Caprines | EQ21_and_Caprines79 | -6.36583332 | -4.50157 | 0.000976 | -8.8E-05 |
| 187 | EQ21_and_Caprines | EQ21_and_Caprines80 | -5.3656303 | -3.8206 | 0.00079 | -9.4E-05 |
| 188 | EQ21_and_Caprines | EQ21_and_Caprines81 | -5.24238852 | -1.98765 | 0.000637 | -2.6E-05 |
| 189 | EQ21_and_Caprines | EQ21_and_Caprines82 | -5.2423885 | -1.98765 | 0.000653 | -2.3E-05 |
| 190 | EQ21_and_Caprines | EQ21_and_Caprines83 | -4.99800631 | -1.24893 | 0.000445 | 1.32E-05 |
| 191 | EQ21_and_Caprines | EQ21_and_Caprines84 | -5.37931738 | -0.36323 | 0.000484 | 0.000183 |
| 192 | EQ21_and_Caprines | EQ21_and_Caprines85 | -5.95586136 | -0.66392 | 0.000744 | 0.000155 |
| 193 | EQ21_and_Caprines | EQ21_and_Caprines86 | -6.3324232 | -0.04854 | 0.000879 | 0.000318 |
| 194 | EQ21_and_Caprines | EQ21_and_Caprines87 | -1.74154434 | 0.17021 | -0.00066 | 0.000276 |
| 195 | EQ21_and_Caprines | EQ21_and_Caprines88 | -1.23058412 | 0.967547 | -0.00082 | 0.000315 |
| 196 | EQ21_and_Caprines | EQ21_and_Caprines89 | -0.73704636 | 0.861502 | -0.0009 | 0.000376 |
| 197 | EQ21_and_Caprines | EQ21_and_Caprines90 | -0.46297939 | 0.740208 | -0.0009 | 0.000329 |
| 198 | EQ21_and_Caprines | EQ21_and_Caprines91 | -1.11313857 | -1.29977 | -0.00026 | 5.65E-06 |
| 199 | EQ21_and_Caprines | EQ21_and_Caprines92 | 0.13060381 | 0.420585 | -0.00076 | 7.43E-05 |
| 200 | EQ21_and_Caprines | EQ21_and_Caprines93 | -0.14216011 | -1.58314 | -0.00014 | -0.00018 |
| 201 | EQ21_and_Caprines | EQ21_and_Caprines94 | -1.50151595 | -2.51962 | 6.19E-05 | -9.4E-05 |
| 202 | EQ21_and_Caprines | EQ21_and_Caprines95 | -1.95527147 | -2.84185 | 0.000158 | -7.7E-05 |
| 203 | EQ21_and_Caprines | EQ21_and_Caprines96 | -1.76436202 | -0.94405 | -0.00034 | 0.00013 |
| 204 | EQ21_and_Caprines | EQ21_and_Caprines97 | -1.61264427 | 1.171554 | -0.00079 | 0.000137 |
| 205 | EQ21_and_Caprines | EQ21_and_Caprines98 | -1.35350839 | 0.918909 | -0.0008 | 0.000296 |
| 206 | EQ21_and_Caprines | EQ21_and_Caprines99 | -1.42878686 | 0.602931 | -0.00079 | 0.000336 |
| 207 | EQ21_and_Caprines | EQ21_and_Caprines100 | -1.79572329 | -0.435 | -0.00048 | 0.000214 |
| 208 | EQ21_and_Caprines | EQ21_and_Caprines101 | -2.33048982 | -1.16382 | -0.00019 | 0.000157 |
| 209 | EQ21_and_Caprines | EQ21_and_Caprines102 | -3.33083297 | -2.50639 | 0.000288 | -9.7E-06 |
| 210 | EQ21_and_Caprines | EQ21_and_Caprines103 | -4.04311557 | -2.55589 | 0.00043 | -0.00022 |
| 211 | EQ21_and_Caprines | EQ21_and_Caprines104 | -3.48804556 | -2.30681 | 0.000281 | -2E-06 |
| 212 | EQ21_and_Caprines | EQ21_and_Caprines105 | -1.99770924 | -2.53405 | 0.000102 | -2.9E-05 |
| 213 | EQ21_and_Caprines | EQ21_and_Caprines106 | -3.86841805 | -4.83927 | 0.00068 | -0.00011 |
| 214 | EQ21_and_Caprines | EQ21_and_Caprines107 | -3.6668196 | -4.85533 | 0.000658 | -0.00011 |
| 215 | EQ21_and_Caprines | EQ21_and_Caprines108 | -3.81302148 | -4.19388 | 0.000589 | -9E-05 |
| 216 | EQ21_and_Caprines | EQ21_and_Caprines109 | -3.94813263 | -3.69204 | 0.000546 | -7.6E-05 |
| 217 | EQ21_and_Caprines | EQ21_and_Caprines110 | -3.71054636 | -2.78338 | 0.000389 | -2.8E-05 |
| 218 | EQ21_and_Caprines | EQ21_and_Caprines111 | -4.14014064 | -0.9287 | 0.000111 | 0.000101 |
| 219 | EQ21_and_Caprines | EQ21_and_Caprines112 | 8.80929495 | -2.63519 | 0.001011 | 0.000864 |
| 220 | EQ21_and_Caprines | EQ21_and_Caprines113 | 9.19383325 | -3.84143 | 0.001236 | 0.00086 |
| 221 | EQ21_and_Caprines | EQ21_and_Caprines114 | 7.47510457 | -5.07354 | 0.001523 | -0.00031 |
| 222 | EQ21_and_Caprines | EQ21_and_Caprines115 | 4.77189451 | -5.12103 | 0.001627 | -0.00146 |
| 223 | EQ21_and_Caprines | EQ21_and_Caprines116 | -5.03445298 | -2.48443 | 0.000635 | -3.8E-05 |
| 224 | EQ21_and_Caprines | EQ21_and_Caprines117 | -5.93436764 | -2.51689 | 0.000823 | -4.5E-05 |
| 225 | EQ21_and_Caprines | EQ21_and_Caprines118 | -7.31927313 | -1.34005 | 0.001169 | 0.000126 |
| 226 | EQ21_and_Caprines | EQ21_and_Caprines119 | -3.66361782 | -1.72759 | 0.000208 | 6.54E-05 |
| 227 | EQ21_and_Caprines | EQ21_and_Caprines120 | -3.66361779 | -1.72759 | 0.000188 | 6.12E-05 |
| 228 | EQ21_and_Caprines | EQ21_and_Caprines121 | -2.09862285 | 1.428486 | -0.00077 | 3.72E-05 |
| 229 | EQ21_and_Caprines | EQ21_and_Caprines122 | -1.83793411 | -3.76594 | 0.000332 | -0.00019 |
| 230 | EQ21_and_Caprines | EQ21_and_Caprines123 | -1.27946703 | -6.03268 | 0.000748 | -0.00041 |
| 231 | EQ21_and_Caprines | EQ21_and_Caprines124 | 0.00897388 | -2.93592 | 0.000204 | -0.00042 |
| 232 | EQ21_and_Caprines | EQ21_and_Caprines125 | -1.88524056 | 1.24283 | -0.0008 | 9.77E-05 |
| 233 | EQ21_and_Caprines | EQ21_and_Caprines126 | -2.37289031 | -0.39491 | -0.00038 | 0.000249 |
| 234 | EQ21_and_Caprines | EQ21_and_Caprines127 | -2.89098241 | -3.03718 | 0.000302 | -4.4E-05 |
| 235 | EQ21_and_Caprines | EQ21_and_Caprines128 | -2.3949007 | -4.40609 | 0.00048 | -0.00019 |
| 236 | EQ21_and_Caprines | EQ21_and_Caprines129 | -3.32739442 | -3.96968 | 0.000503 | -0.00011 |
| 237 | EQ21_and_Caprines | EQ21_and_Caprines130 | -3.27344118 | -1.86086 | 0.000165 | 5.82E-05 |
| 238 | EQ21_and_Caprines | EQ21_and_Caprines131 | -2.87861927 | -0.32981 | -0.00033 | 0.000258 |
| 239 | EQ21_and_Caprines | EQ21_and_Caprines132 | -2.11941786 | 1.727764 | -0.00078 | -6.6E-05 |
| 240 | EQ21_and_Caprines | EQ21_and_Caprines133 | -3.66283159 | -0.58478 | -0.00011 | 0.00017 |
| 241 | EQ21_and_Caprines | EQ21_and_Caprines134 | -2.89248264 | -0.13029 | -0.0004 | 0.000282 |
| 242 | EQ21_and_Caprines | EQ21_and_Caprines135 | -2.40061704 | 0.004126 | -0.0005 | 0.000284 |
| 243 | EQ21_and_Caprines | EQ21_and_Caprines136 | -2.48651313 | -0.20233 | -0.00042 | 0.00027 |
| 244 | EQ21_and_Caprines | EQ21_and_Caprines137 | -2.17608708 | -1.78461 | -1.2E-05 | 9.38E-05 |
| 245 | EQ21_and_Caprines | EQ21_and_Caprines138 | -2.17608716 | -1.78461 | -5.1E-05 | 6.11E-05 |
| 246 | EQ21_and_Caprines | EQ21_and_Caprines139 | -0.96511007 | -1.90095 | -8.6E-05 | -0.00014 |
| 247 | EQ21_and_Caprines | EQ21_and_Caprines140 | -0.82526071 | 0.414311 | -0.00103 | -0.00017 |
| 248 | EQ21_and_Caprines | EQ21_and_Caprines141 | 0.25823023 | 0.69008 | -0.00066 | -7.8E-05 |
| 249 | EQ21_and_Caprines | EQ21_and_Caprines142 | 0.25823027 | 0.69008 | -0.00065 | -6.3E-05 |
| 250 | EQ21_and_Caprines | EQ21_and_Caprines143 | -0.11579667 | -1.14025 | -0.00025 | -0.0001 |
| 251 | EQ21_and_Caprines | EQ21_and_Caprines144 | -1.47083916 | -1.83585 | -0.0001 | -1.6E-05 |
| 252 | EQ21_and_Caprines | EQ21_and_Caprines145 | -3.26922514 | -3.36419 | 0.000418 | -5E-05 |
| 253 | EQ21_and_Caprines | EQ21_and_Caprines146 | -3.26922514 | -3.36419 | 0.000384 | -5.7E-05 |
| 254 | EQ21_and_Caprines | EQ21_and_Caprines147 | -2.57934091 | -0.30902 | -0.00037 | 0.000262 |
| 255 | EQ21_and_Caprines | EQ21_and_Caprines148 | -2.39911679 | -2.90276 | 0.000215 | -3.7E-05 |
| 256 | EQ21_and_Caprines | EQ21_and_Caprines149 | -2.46028618 | 2.305527 | -0.00097 | -4E-05 |
| 257 | EQ21_and_Caprines | EQ21_and_Caprines150 | -1.45576011 | 2.275084 | -0.00099 | -0.00035 |
| 258 | EQ21_and_Caprines | EQ21_and_Caprines151 | -1.97778333 | -6.0812 | 0.000751 | -0.0003 |
| 259 | EQ21_and_Caprines | EQ21_and_Caprines152 | -2.8493922 | -3.63574 | 0.000394 | -0.0001 |
| 260 | EQ21_and_Caprines | EQ21_and_Caprines153 | -3.7002058 | -1.48955 | 0.000138 | 5.21E-05 |
| 261 | EQ21_and_Caprines | EQ21_and_Caprines154 | -3.83883938 | 0.505639 | -0.0003 | 0.000266 |
| 262 | EQ21_and_Caprines | EQ21_and_Caprines155 | -3.06155883 | 0.860371 | -0.00044 | 0.00022 |
| 263 | EQ21_and_Caprines | EQ21_and_Caprines156 | -2.36324276 | 0.908893 | -0.00065 | 0.000216 |
| 264 | EQ21_and_Caprines | EQ21_and_Caprines157 | -2.34666339 | 2.112939 | -0.00088 | -8.9E-05 |
